# Understanding the impact of covariates on the classification of implementation units for soil-transmitted helminths control: a case study from Kenya

**DOI:** 10.1186/s12874-024-02420-1

**Published:** 2024-11-29

**Authors:** Amitha Puranik, Peter J. Diggle, Maurice R. Odiere, Katherine Gass, Stella Kepha, Collins Okoyo, Charles Mwandawiro, Florence Wakesho, Wycliff Omondi, Hadley Matendechero Sultani, Emanuele Giorgi

**Affiliations:** 1 Centre for Health Informatics, Computing, and Statistics (CHICAS), Lancaster Medical School, Lancaster University, Lancaster, United Kingdom; 2 Centre for Global Health Research, Kenya Medical Research Institute, Kisumu, Kenya; 3 Neglected Tropical Diseases Support Center (NTD-SC), Task Force for Global Health, Atlanta, USA; 4 Division of Vector Borne and Neglected Tropical Diseases, Ministry of Health, Nairobi, Kenya; 5 Eastern and Southern Africa Centre of International Parasite Control (ESACIPAC), Kenya Medical Research Institute, Nairobi, Kenya; 6 Department of Epidemiology, Statistics and Informatics (DESI), Kenya Medical Research Institute, Nairobi, Kenya; 7 State Department for Public Health and Professional Standards, Ministry of Health, Nairobi, Kenya

**Keywords:** Soil-transmitted helminthiasis, Geostatistical methods, Spatially referenced covariates, STH transmission classes, Kenya

## Abstract

**Background:**

Soil-transmitted helminthiasis (STH) are a parasitic infection that predominantly affects impoverished regions. Model-based geostatistics (MBG) has been established as a set of modern statistical methods that enable mapping of disease risk in a geographical area of interest. We investigate how the use of remotely sensed covariates can help to improve the predictive inferences on STH prevalence using MBG methods. In particular, we focus on how the covariates impact on the classification of areas into distinct class of STH prevalence.

**Methods:**

This study uses secondary data obtained from a sample of 1551 schools in Kenya, gathered through a combination of longitudinal and cross-sectional surveys. We compare the performance of two geostatistical models: one that does not make use of any spatially referenced covariate; and a second model that uses remotely sensed covariates to assist STH prevalence prediction. We also carry out a simulation study in which we compare the performance of the two models in the classifications of areal units with varying sample sizes and prevalence levels.

**Results:**

The model with covariates generated lower levels of uncertainty and was able to classify 88 more districts into prevalence classes than the model without covariates, which instead left those as “unclassified”. The simulation study showed that the model with covariates also yielded a higher proportion of correct classification of at least 40% for all sub-counties.

**Conclusion:**

Covariates can substantially reduce the uncertainty of the predictive inference generated from geostatistical models. Using covariates can thus contribute to the design of more effective STH control strategies by reducing sample sizes without compromising the predictive performance of geostatistical models.

**Supplementary Information:**

The online version contains supplementary material available at 10.1186/s12874-024-02420-1.

## Background

Soil-transmitted helminthiasis (STH) is a widespread human parasite infection that primarily affects the poorest and most destitute areas, with the highest burden of the disease in sub-Saharan Africa, Southeast Asia, and the Americas. STH is estimated to affect over 1.5 billion people across the globe, over 914 million of whom are children. Globally, the most common species of STH that infect people are *Ascaris lumbricoides* (roundworm), *Trichuris trichiura* (whipworm), *Ancylostoma duodenale* and *Necator americanus* (hookworm). These species, which are the focus of this paper, are typically treated as a group because they can be detected using the same diagnostic procedures and respond similarly to the same medications [1].

More than six million school-age children in Kenya are at risk of parasitic worms, including STH infections [2]. The transmission of STH occurs predominantly through the exposure to contaminated water, food, and soil. The factors that contribute to the high prevalence of STH are substandard hygiene practices, poor sanitation, and limited availability of safe water sources. Children playing in contaminated soil or walking barefoot on ground that is contaminated are highly susceptible to acquiring STH infections [3]. The Kenyan government and their international partners have implemented a series of measures to effectively manage and control the spread of STH infections. The national school-based deworming programme (NSBDP) focuses on administering mass drug administration (MDA) to school-aged children, resulting in a notable decrease in the prevalence of STH infections [4]. In addition to MDA, the government has been conducting health education campaigns to promote good sanitation and hygiene practices, such as washing of hands with soap and water after using the toilet and before eating [5]. Priority has also been given to enhancing sanitation through the construction of latrines and the supply of clean water sources [6].

Model-based geostatistical (MBG) methods have been shown to enhance predictive inferences on prevalence by delivering desired levels of precision at smaller sample sizes than standard sampling designs [7, 8]. MBG models allow the inclusion of spatially referenced covariates to assist the prediction of disease prevalence at unsampled locations [9]. Previous work on the mapping of STH prevalence has made use of environmental and socioeconomic covariates, as well as indicators of improved access to sanitation facilities, and drinking water facilities [10–14]. Whilst the scientific rationale for the inclusion of these covariates is well established, the statistical advantages that are accrued from their use have not been fully investigated.

In previous geostatistical analysis of STH in Cambodia [15], three geostatistical models were fitted: a model without covariates; a model including only environmental variables; a model including environmental, demographic, and behavioural covariates. The three models were only compared in terms of the changes of the scale of the residual spatial correlation in prevalence; no evaluation was made of the additional predictive power obtained by the inclusion of covariates. In another study focused on the geostatistical mapping of *Strongyloides* prevalence, a model without covariates was compared to one that includes covariates, but only in terms of the reduction of the deviation information criterion [16]. Especially in low prevalence settings, estimated regression relationships between risk factors and prevalence are highly imprecise. For this reason, models without covariates often provide pragmatic solutions [9]. According to our knowledge, no research has been conducted to assess the impact and usefulness of spatially referenced covariates to classify areas into different classes of STH transmission using geostatistical methods.

In this paper we investigated the usefulness of covariates to classify areal units into pre-defined STH prevalence classes, using MBG methods. Our focus was on modelling the combined prevalence of any of *Ascaris*, *Trichuris* and hookworm infections, which is currently used in the guidelines of the World Health Organization (WHO) to target the rollout and determine the frequency of MDA [17]. Through a case study from Kenya and a simulation study, we quantified the additional benefits of using covariates for the correct classification of spatial units used for policy decision-making and how these can be used to reduce the sample size without compromising the predictive performance of the model.

## Material and methods

### Study area

Kenya stretches from latitudes − 4.472 to 3.937 and from longitudes 33.976 to 41.857 (Additional file 1: Figure S1). The country is 582,646 km² in area. Kenya borders with Tanzania in the South, Uganda in the West, South Sudan in the Northwest, Ethiopia in the North and Somalia in the East. As per Census 2019, the enumerated population for the country was recorded at 47,564,296 [18].

### Data description

In what follows, we shall use the term “county” to refer to the geographical units created by the 2010 Constitution of Kenya as the new units of devolved government. The data included the number of children tested and the number of children who tested positive for any species of STH, in 1551 schools of Kenya selected from 23 counties. Out of these counties, a total of 20 have received all six scheduled rounds of MDA [19]. The geographical location of each school was recorded as latitude and longitude. The data was retrieved from the longitudinal survey conducted by the Kenya NSBDP for the year 2017 (January, February, May) [4] and the year 2018 (January, February, May) [20] and from a cross-sectional survey conducted at a fine spatial scale (September 2021, Division of Vector Borne and Tropical Neglected Diseases under Ministry of Health Kenya, unpublished data). The NSBDP data consists of surveys conducted pre and post intervention in primary schools with an average of 108 children enrolled from each school, before and immediately after the deworming activity to evaluate reductions in STH infections that can be directly attributed to programme implementation [21]. For the granular mapping, cross-sectional surveys were conducted among school-age children in 5 primary schools selected in each mapping unit (ward), with an average of 60 children enrolled from each school. In both these surveys, stool samples were collected, and prevalence and intensity of STH were determined using the Kato-Katz technique [19].

In the supplementary material (Additional file 1: Figure S1), we show the map of the sampled locations for the South-West and South-East regions of Kenya. The prevalence of STH was distinct between the two regions, as were their ecological characteristics. Hence the two regions were studied separately. The Western region (pink) consisted of 22 counties, 142 sub-counties, and 875 schools, whilst the Eastern region (orange) consisted of 20 counties, 122 sub-counties, and 618 schools, after the exclusion of 38 schools that belonged to Lamu Archipelago of Lamu county and 20 schools that did not have STH data.

### Statistical analysis

#### Selection of covariates

The lists of environmental and socio-economic variables considered in the study are provided in Additional file 1: Table S1. We carry out the selection into two steps: an initial step that discard covariates that show a very weak correlation; a more formal selection using a Binomial mixed model. The rationale for this is that the first step helps to reduce the number of variables to consider in the second step and alleviate the computational burden. The initial selection of covariates was carried out by excluding variables that were highly correlated (correlation coefficient > 0.8) with other variables or that showed a correlation coefficient less than 0.2 with the empirical logit of STH prevalence. We point out that the empirical logit was used only up to this stage of the exploratory analysis. After this first selection, a Binomial mixed model with a location specific random intercept was fitted and variables that were not found to be significant at the 5% conventional level of significance were excluded (see Additional file 1: Table S2). The remaining variables were then provisionally included in the geostatistical model (see next section) but, if found statistically non-significant, were then sequentially discarded from the model, starting from the variable showing the largest *p*-value.

#### Geostatistical model formulation, parameter estimation and spatial prediction

For $\:i=1,\dots\:n$, let $\:{Y}_{i}$denote the number of children out of $\:{m}_{i}$ tested children, who tested positive for at least one of the three STH species at a sampled school with location $\:{x}_{i}$ and $\:d\left({x}_{i}\right)$ the vector of covariates at location $\:{x}_{i}$ that have been identified in the selection process described in the previous section. Let $\:S\left({x}_{i}\right)$ denote a stationary and isotropic Gaussian process and $\:{Z}_{i}$ a set of mutually independent mean-zero Gaussian variables. Conditionally on $\:S\left({x}_{i}\right)$ and $\:{Z}_{i}$, the $\:{Y}_{i}$ are assumed to be mutually independent Binomial random variables with probabilities $\:p\left({x}_{i}\right)$, representing the STH prevalence at location $\:{x}_{i}$. We then model $\:p\left({x}_{i}\right)$ using a mixed effects logistic regression taking the form

$$\:\text{log}\left\{\frac{p\left({x}_{i}\right)}{1-p\left({x}_{i}\right)}\right\}=d{\left({x}_{i}\right)}^{{\prime\:}}\beta\:+S\left({x}_{i}\right)+\:{Z}_{i}$$

We assume an exponential spatial correlation function for the spatial process $\:S\left({x}_{i}\right)$, hence $\:cov\left\{S\left({x}_{i}\right),S\left({x}_{j}\right)\right\}={\sigma\:}^{2}exp\{-{u}_{ij}/\phi\:\}$, where $\:{u}_{ij}$ is the distance between $\:{x}_{i}$ and $\:{x}_{j}$, $\:{\sigma\:}^{2}$ is the variance of $\:S\left({x}_{i}\right)$, $\:\phi\:$ is the scale parameter of the spatial correlation and we denote the variance of the $\:{Z}_{i}$ by $\:{\tau\:}^{2}$. Based on an exploratory analysis, all covariates were assumed to have a linear relationship on the log-odds scale (Additional file 1: Figure S3a and Figure S3b).

#### Spatial prediction of STH prevalence and endemicity classes at pixel and sub-county- level

For the spatial predictions of STH prevalence at pixel-level, a 4.5 by 4.5 km regular pixel grid was created for each subcounty. Pixels with a population density less than 10 were excluded to avoid carrying out predictions in sparsely populated areas for which the data provide only limited information.

A geostatistical binomial logistic model was fitted using the MCML estimation method implemented in the PrevMap R package [22]. Spatial prediction was carried out using plug-in maximum likelihood estimates. The mean and standard error of the predictive distribution were obtained for each pixel [22].

We also generated the predictive probabilities of belonging to prespecified prevalence classes at pixel-level. Based on the STH decision tree by WHO, after five years of yearly or twice-yearly preventive chemotherapy in areas with STH prevalence between 20% and 50% or above 50%, respectively, the MDA protocol must be sustained based on the categories pertaining to endemicity that are determined by thresholds for prevalence as follows:

suspend MDA if STH prevalence is less than 2%; perform MDA every two years if STH prevalence is between 2% and 10%; annually if prevalence is between 10% and 20%; twice-yearly if between 20% and 50%; three times per year if above 50%. We generated maps for the predictive probability that each pixel belongs to each of these prevalence classes [17].

We also calculated predictive probabilities for membership to the same set of prevalence classes at sub-county level, as follows.

1. Generate predictive samples from the joint predictive distribution of prevalence for each pixel falling within the given sub-county.
2. Average over the prevalence samples across each pixel, weighted by population density.
3. Compute the probability of membership to each endemicity class (2%, 2%−10%, 10%−20%, 20%−50% and above 50%) as the proportion of weighted average samples that fall within that class.

The above steps were performed using models without and with covariates.

#### Simulation study

A simulation study allows us to compare the predictive inferences of the model against the true simulated prevalence under pre-defined scenarios. Here, a simulation study was carried out to gain a better understanding of the impact on spatial prediction resulting from: (1) model specification (2) average prevalence (3) number of sampling locations. Data was simulated for the observed locations in the Western and Eastern regions of Kenya and for prediction locations using the true model $\:{M}_{T}$, from which the simulated data were generated, and which included the four covariates each identified from Additional file 1: Table S2. The performance of the true model was compared with $\:{M}_{I}$, a model which does not use any covariates. The two scenarios of the simulation were obtained by varying i) the average prevalence of STH and ii) the number of sampled locations. Table 1 shows the full set of scenarios considered in the simulation. Note that 875 and 618 are the numbers of locations of the original data in the Western and Eastern regions, respectively.

**Table 1:** Specifications of data simulation

Scenario	Sample size	Prevalence (%)	Region
S1	875	6.5	Western
	618	1	Eastern
S2	875	12	Western
	618	10	Eastern
S3	438	6.5	Western
	309	1	Eastern
S4	438	12	Western
	309	10	Eastern

Each simulation proceeds through the following iterative steps.

1. Run a simulation to determine the true prevalence using $\:{M}_{T}$ at the observed locations and over a regular grid at 4.5 by 4.5 km spacing to cover each sub-county.
2. Simulate a binomial dataset for the number of STH cases at the observed locations, using the simulated prevalence from the previous step.
3. For each of the two scenarios, fit the $\:{M}_{T}$ and $\:{M}_{I}$ models to the simulated data set.
4. Generate subcounty-level spatial predictions for prevalence by averaging over the 4.5 by 4.5 km regular grid weighted by population density, for each of the two fitted models.
5. Repeat steps 1 to 4, 1000 times.

Upon completion of these steps, the performance of $\:{M}_{T}$ and $\:{M}_{I}$ in each scenario was summarized at sub-county level based on the proportion of correct classifications. To quantify the correct classification (%), the average of each predicted sample weighted for population density was obtained and the predictive probabilities for each of the ranges less than 2%, 2%−10%, 10%−20%, 20%−50% and 50% and above, were computed. The predicted class was computed as the range of prevalence for which the model provides the largest predictive probability. We compute the proportion of sub-counties correctly classified across the 1000 simulations by computing the number of times that the predicted and true prevalence classes matched.

## Results

### Spatial correlation and associated factors of STH

The crude prevalence of STH was 6.5% in the Western region and 1% in the Eastern region. The empirical variogram (Additional file 1: Figure S2) showed evidence of residual spatial correlation after fitting a model with covariates, thus justifying the use of a geostatistical model to map STH prevalence. In the Western region, the covariates identified through the variable selection process were aridity index, enhanced vegetation index (monthly average), tasseled cap wetness (monthly average), and availability of improved drinking water source from Water, Sanitation and Hygiene (WASH). For the Eastern region, the selected covariates were aridity index, enhanced vegetation index (monthly average), rainfall (monthly average) and elevation.

The Western (Table 2) and Eastern (Table 3) regions both indicated a substantially larger estimates of the $\:{\sigma\:}^{2}$ in comparison to $\:{\tau\:}^{2}$, which suggested the presence of a strong residual spatial variation in the data. This was the case in both models without and with covariates.

The estimated values of φ for the two regions imply that the spatial correlation reached a value of 0.05 at 84.9 km and 120.3 km in the Western and Eastern regions, respectively, from the model without covariates. In the model with covariates, these values were reduced to 37.1 km and 22.8 km.

In the estimates reported in Table 2 and Table 3, we observe that the point estimates for $\:{\sigma\:}^{2}$ substantially decreased after the introduction of the covariates in both regions. In the Western region, the estimated value in the model with covariates was about half of the model without, and for the Eastern region, the point estimate decreased by about a third. These results suggested that the covariates played a significant role in explaining the residual spatial variation and thus would be expected to reduce the uncertainty in our predictive inferences.

**Table 2:** MCML estimates, standard errors, 95% confidence intervals for Binomial geostatistical models - Western region Kenya

	Estimate	95% Confidence interval	
		Lower limit	Upper limit
*Model without covariates*			
Intercept	−2.612	−3.253	−1.971
$\sigma^{2}$	1.140	0.634	2.048
φ	28.304	14.004	57.205
$\tau^{2}$	0.313	0.092	1.067
*Model with covariates*			
Intercept	−3.251	−4.925	−1.577
Aridity index	1.508	0.046	2.970
Enhanced vegetation index - monthly average	5.497	2.416	8.578
Tasseled cap wetness - monthly average	10.056	4.744	15.368
WASH – availability of improved drinking water	−1.435	−2.574	−0.296
$\sigma^{2}$	0.503	0.318	0.795
φ	12.367	5.313	28.782
$\tau^{2}$	0.319	0.117	0.873

**Table 3:** MCML estimates, standard errors, 95% confidence intervals for Binomial geostatistical models - Eastern region Kenya

	Estimate	95% Confidence interval	
		Lower limit	Upper limit
*Model without covariates*			
Intercept	−4.899	−5.918	−3.880
$\sigma^{2}$	3.047	1.583	5.863
φ	40.085	15.862	101.299
$\tau^{2}$	0.694	0.181	2.650
*Model with covariates*			
Intercept	−6.699	−7.811	−5.587
Aridity index	4.275	2.065	6.485
Enhanced vegetation index - monthly average	6.271	3.283	9.259
Rainfall- monthly average	−0.017	−0.029	−0.004
Elevation	−0.002	−0.002	−0.001
$\sigma^{2}$	0.982	0.615	1.567
φ	7.598	3.315	17.412
$\tau^{2}$	0.583	0.172	1.978

### Spatial prediction of STH

**Fig. 1:**
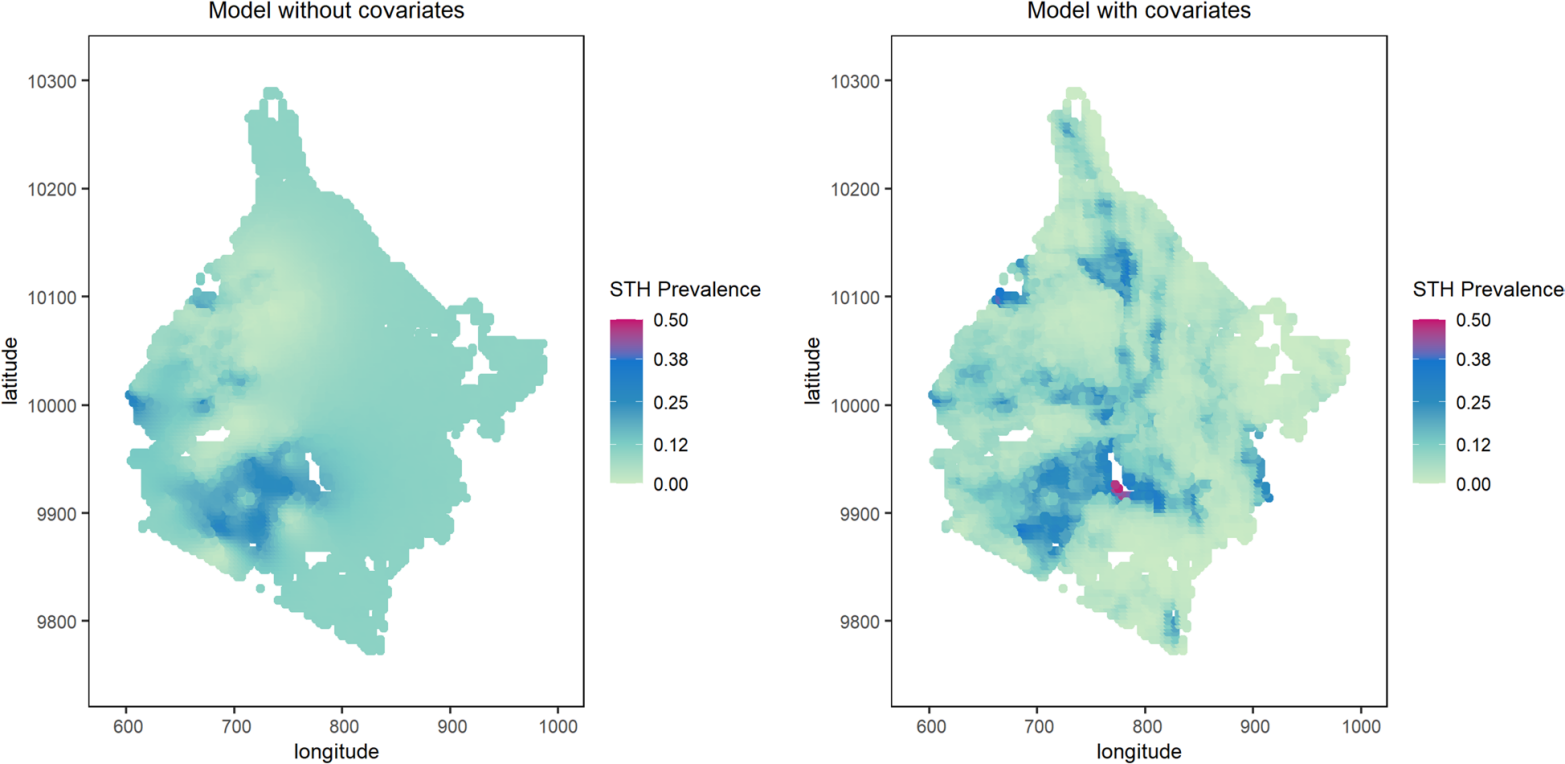
Predicted prevalence of STH obtained from geostatistical models in Western region of Kenya

**Fig. 2:**
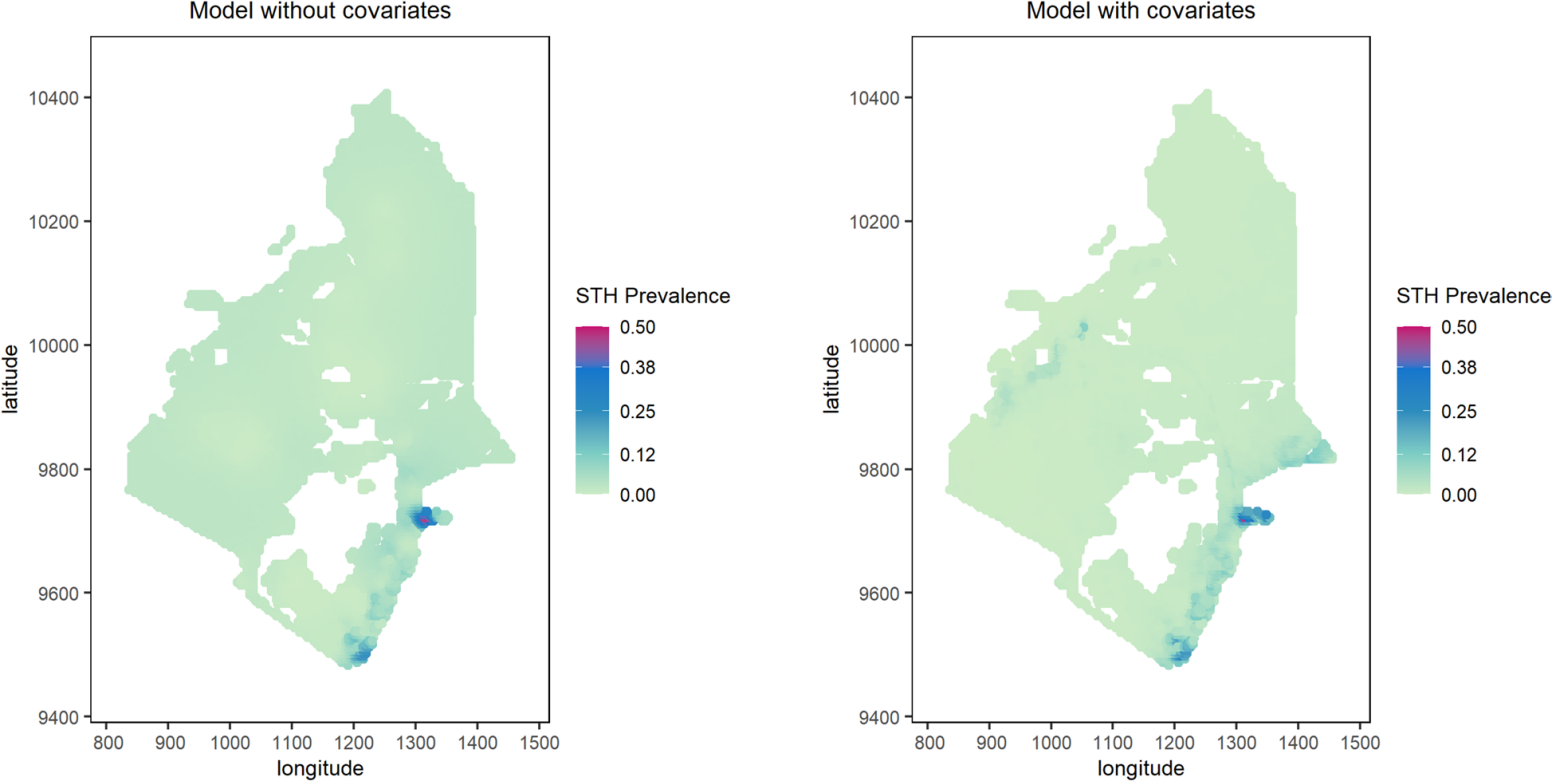
Predicted prevalence of STH obtained from geostatistical models in Eastern region of Kenya

The maps of predicted prevalence of STH are provided in Fig. 1 and Fig. 2 for the Western and Eastern regions, respectively, obtained from the geostatistical model without and with covariates. The maps of standard errors of predicted prevalence are given in Additional file 1: Figure S4 and Additional file 1: Figure S5 and the average standard errors of predicted prevalence of STH is in Additional file 1: Table S3.

The model with covariates for the Western region (Fig. 1) delivers more heterogeneous patterns in the predictions of prevalence than the model without covariates, with noticeable discrepancies in the southern part. In the Eastern region, instead, the two models deliver closer predictions with no major discrepancies (Fig. 2).

### Predictive probability maps

**Fig. 3:**
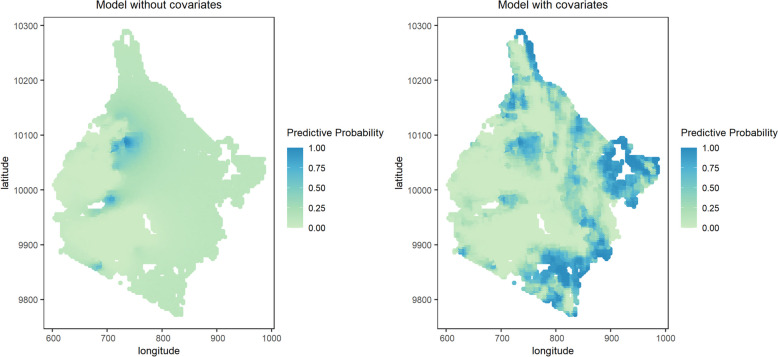
Predictive probability maps for endemicity level of less than 2% for STH from geostatistical models

**Fig. 4:**
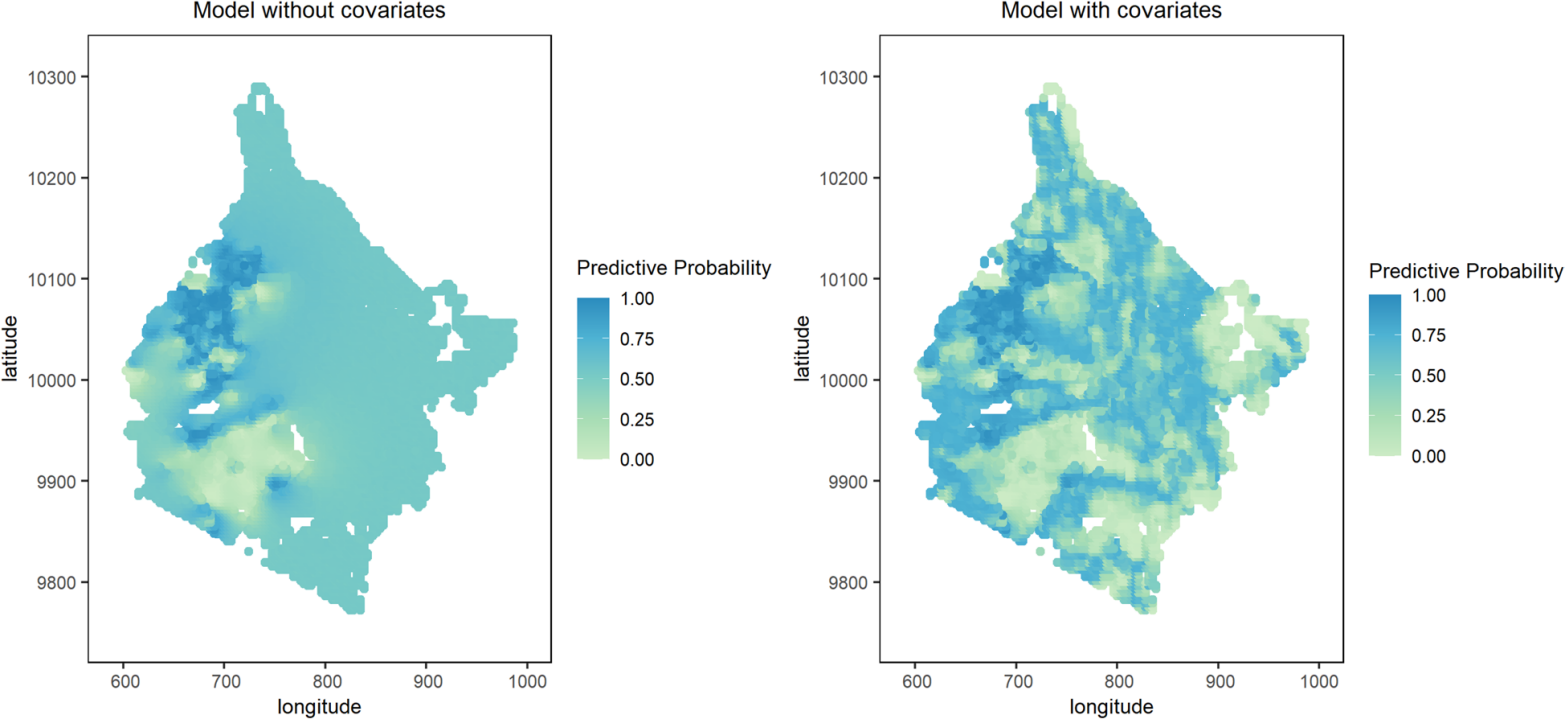
Predictive probability maps for endemicity level between 2% and 10% for STH from geostatistical models

The predictive probability maps for the Western region obtained from the models without and with covariates for endemicity levels of less than 2% and between 2% and 10% are provided in Figs. 3 and 4, respectively. We observe that in areas where data were collected, the two models provide similar answers. In areas where fewer or no data are collected the predictive probabilities used for the classification into prevalence classes often yield different answers. Further results that support this are given by the maps found in the Additional file 1: Figure S6 to Figure S13.

### Prediction of STH prevalence class at sub-county level

**Fig. 5:**
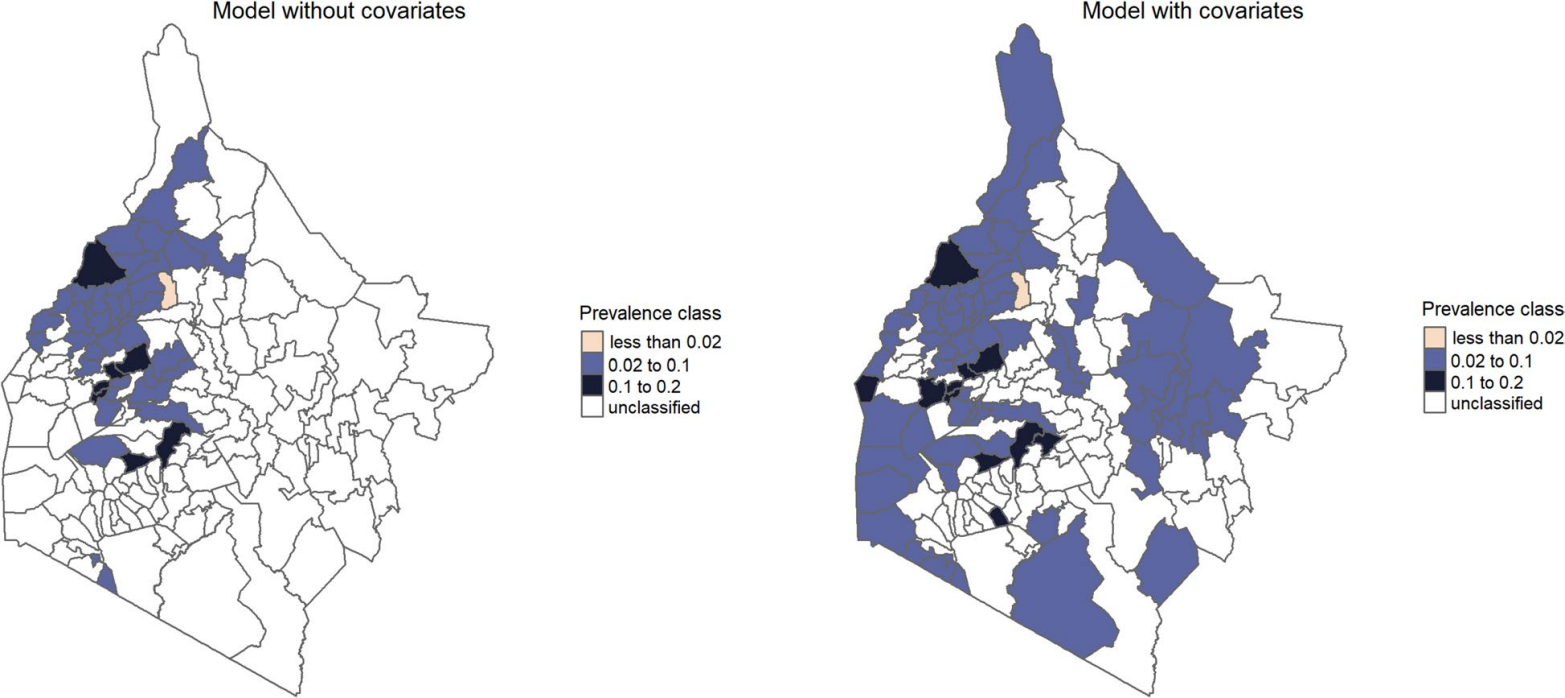
Prevalence class of subcounty for STH with predictive probability greater than 80%: Western region Kenya

**Fig. 6:**
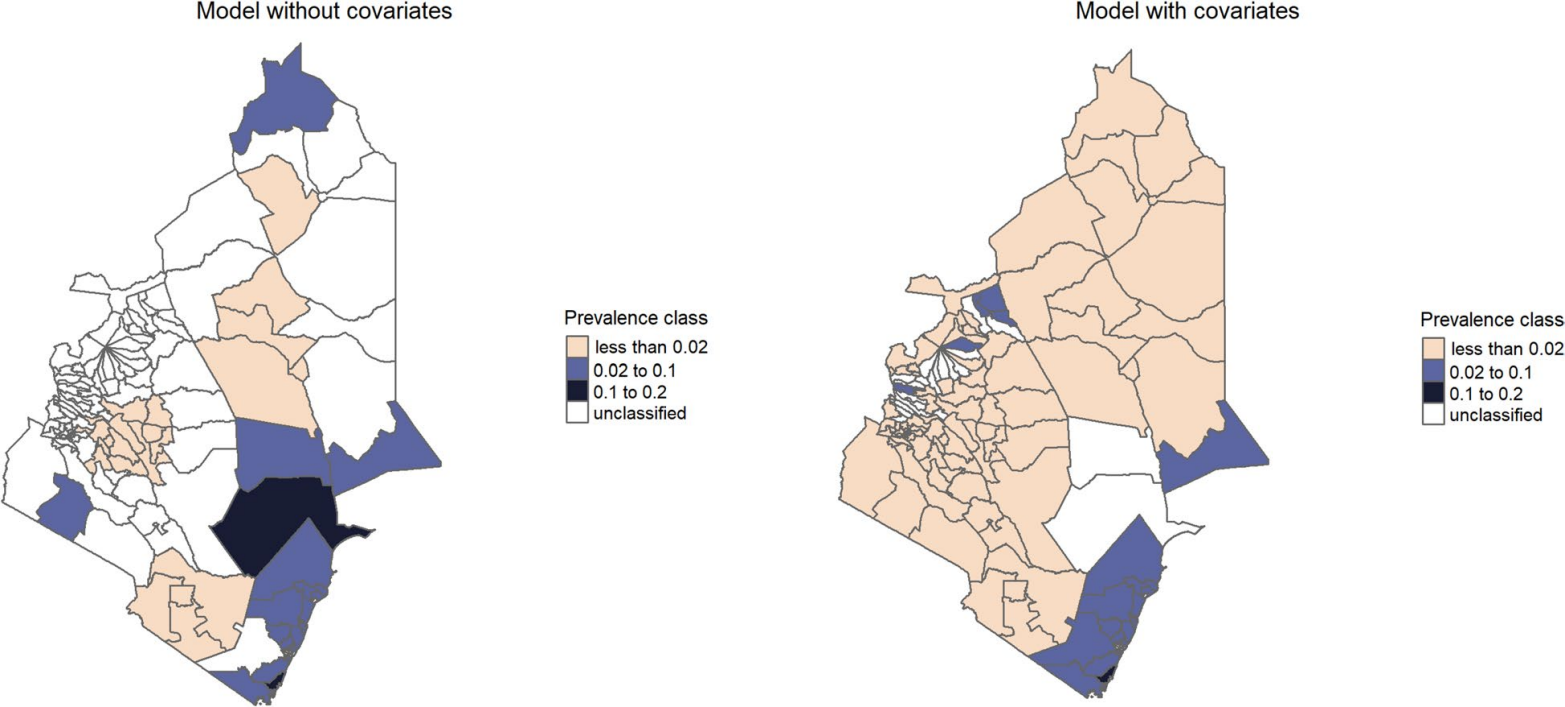
Prevalence class of subcounty for STH with predictive probability greater than 80%: Eastern region Kenya

We present the results of a hypothetical classification of sub-counties, using the exceedance of 80% predictive probability as a rule to allocate each sub-county to a given prevalence class. We point out that the choice of 80% is made only for illustrative purposes and other choices are possible. The value of 80% also corresponds to a relatively high level of certainty that policy makers may consider to use for classifications of aerial units. For a given sub-county, if none of the prevalence classes have at least 80% predictive probability, then the sub-county remains unclassified. The model with covariates correctly classified 51% of sub-counties in the Western region (Fig. 5) and 80% in the Eastern region (Fig. 6), whereas these proportions were 29% (Fig. 5) and 34% (Fig. 6) based on the model without covariates. Table 4 provides a numerical summary of the maps from Figs. 5 and 6.

**Table 4:** Number of subcounties classified to STH prevalence classes with probability greater than 80% using geostatistical models

Region	Model	less than 2%	between 2% and 10%	between 10% and 20%	unclassified
Western region (*n* = 142)	Without covariates	1	33	7	101
	With covariates	1	60	11	70
Eastern region (*n* = 122)	Without covariates	24	15	2	81
	With covariates	80	17	1	24

### Simulation results

**Fig. 7:**
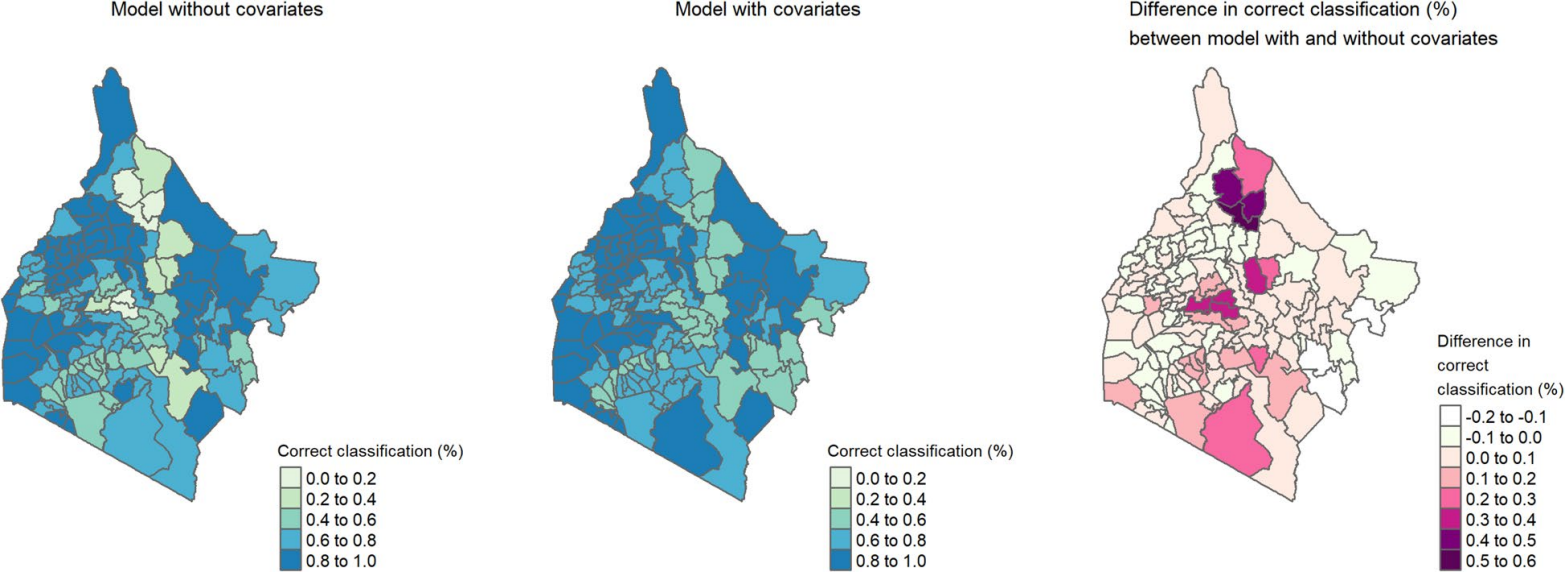
Correct classification (%) at each subcounty for simulated dataset - sample size: 875; average prevalence: 6%

**Fig. 8:**
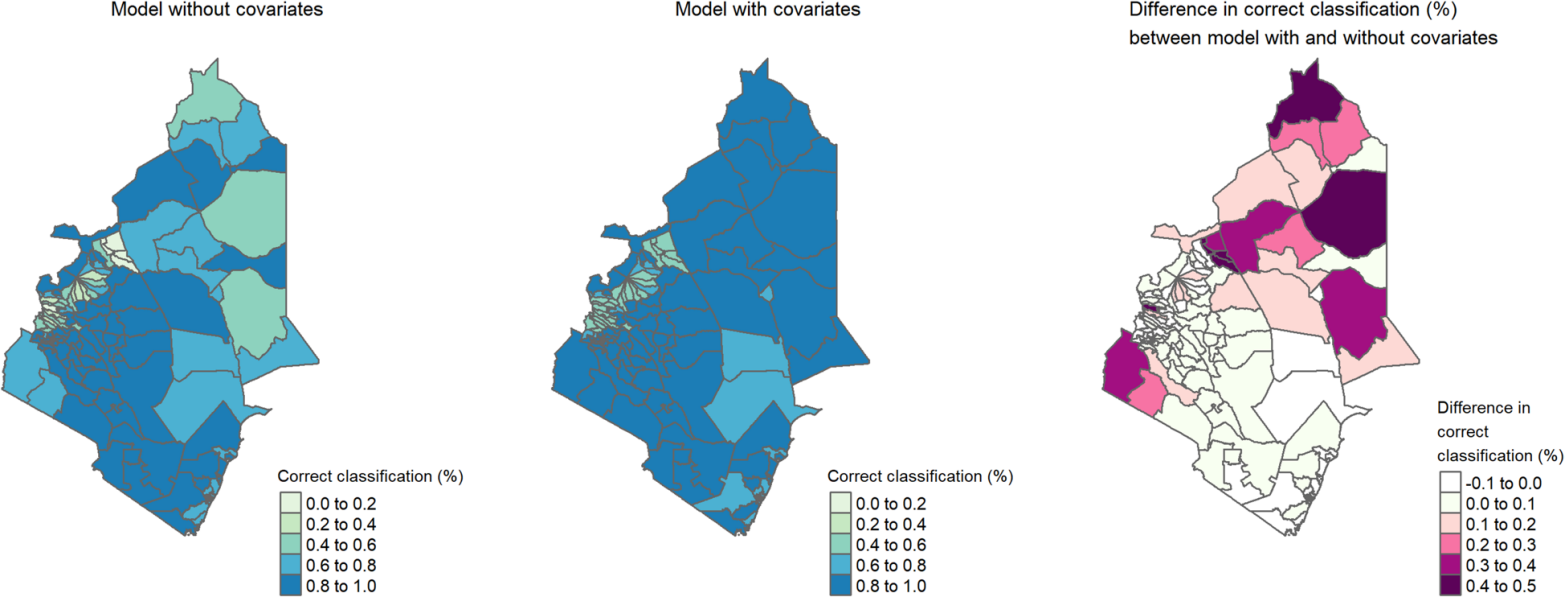
Correct classification (%) at each subcounty for simulated dataset - sample size: 618; average prevalence: 1%

We obtained Figs. 7 and 8 based on the simulation study, which represents the proportion of correct classifications of each sub-county to their respective endemicity levels. The map also provides the difference in proportion of correct classifications between the models with and without covariates. In both the Western and Eastern regions, the model with covariates yielded a correct classification for all the sub-counties of at least 40% and consistently outperformed the model without covariates. More results that show the proportion of correct classification at subcounty-level from the simulation study are provided in Additional file 1: Figure S14 to Figure S19.

## Discussion

This study provides evidence in support of the hypothesis that the inclusion of covariates in a geostatistical model can improve the predictive performance of the model with respect to the classification into ranges of prevalence, both at pixel and sub-county levels. A simulation study was used to quantify the benefits of the inclusion of covariates, since that allows us to generate samples of the true-areal level prevalence and thus compute the probability of correct classification for a given county. Direct evidence of the importance of covariates is also given by the reduction in the variance of the residual spatial random effects, as well as in the standard errors of the predicted prevalence.

The outcome analysed in this was the infection with any species of soil-transmitted helminths. Whilst the most previous work has analysed STH species separately, in this study, we opted for modelling the presence of any species collectively, rather than individually modelling each species, because the species-wise data suffered from numerous missing or incomplete entries which did not allow us to carry out an equally in-depth analysis.

In a simulation study, the expectation is that in principle the data-generating model, in this case the model with covariates, should perform better than a misspecified one, i.e., the model without covariate, however, practical applications often deviate from this expectation [9, 23]. This is because in some cases, the data-generating model may consist of many parameters, which may be difficult to estimate when the amount of data is insufficient. In this case, a simpler model may prove to be more efficient and, surprisingly, could yield superior predictive inferences compared to the more complex data-generating model. Furthermore, even if the data-generating model demonstrates superior performance, the actual gains achieved may be modest or minimal. Consequently, this may weaken the argument for incorporating covariates into the model. The simulation study carried out in this paper allowed us to investigate these aspects while overcome the inherent issues that arise from a simple comparison of the predicted prevalence against the crude observed prevalence [23].

Another finding of our study is that, despite the low overall prevalence of STH in Kenya, the model incorporating covariates was able to classify a larger proportion of sub-counties into specific prevalence classes with a higher degree of confidence compared to the model without covariates. This clearly demonstrates the added value of incorporating these covariates into predictive models for STH prevalence, particularly in regions where the prevalence is low. Both empirical and simulated data exhibited this result.

To the best of our knowledge, this is the first study to report the impact and usefulness of spatially referenced covariates to classify areas into different classes of STH transmission using geostatistical methods. This allows policy makers to make more informed decisions on the administration of MDA programs by locally tailoring the interventions and surveillance, potentially improving the effectiveness of these interventions.

The Western and Eastern regions of Kenya in this study were analysed separately due to their ecological differences. This strategy allowed for a more accurate reflection of the unique environmental and socioeconomic conditions that affect STH prevalence in different parts of the country. The prevalence map developed in our study indicates the heterogeneities associated with STH and that the requirement for MDA is not homogenous throughout the study region, which is important for disease control. Using STH prediction maps to identify sub-regions in need can improve the efficiency of intervention and resource allocation.

The use of the WHO’s STH decision tree [17] provides a structured and globally recognized framework for the classification of STH prevalence classes. The maps of probability for different prevalence thresholds generated from this research provide critical information for policymaking and implementation of MDA strategies. The predictive probabilities reduce the possibility of misclassifying sub-counties and pixels. This ensures that the endemicity levels of STH are accurately determined so that resources are not squandered on interventions and populations that do not need them.

In this study, the spatial predictors for STH that were included in the final model were aridity index, enhanced vegetation index, tasselled cap wetness, availability of improved drinking water source from WASH, rainfall, and elevation. This is in line with findings from previous studies [24–28]. In addition to the covariates considered in this paper, the performance of geostatistical models could be further enhanced by socio-economic variables [29, 30]. When comparing the predictive inferences between the model with covariates against the model without covariates, as expected, this tended to differ the most in areas with fewer or not sampled locations. In addition, predictions from the model with covariates also exhibited a higher spatial heterogeneity than the model without covariates. These differences are explained by the fact that the model without covariates only relies on the Gaussian process for the interpolation of prevalence which reverts to its zero mean in areas that are further away from the data. The validity of the inferences of the model with covariates may also be biased due to the potential misspecifications of the relationship between prevalence and the covariates. However, the use of a linear relationship on the log-odds scale may still adequately capture monotone relationships and, we would expect that, in this case, the negative effects of misspecification would be negligible.

If this analysis were to be conducted in a different country, the same set of initial covariates could be considered, as they represent commonly known environmental and socioeconomic factors associated with soil-transmitted helminth (STH) prevalence. However, we acknowledge that the strength of these associations may differ across countries due to variations in population characteristics, environmental conditions, and the epidemiology of STH. Despite these potential differences, we anticipate that the inclusion of covariates would still be beneficial for reducing predictive uncertainty, even if the specific relationships between covariates and prevalence vary. Our findings suggest that the use of covariates enhances model performance by improving the precision of spatial predictions, and this benefit is likely to be generalizable to other contexts where STH prevalence remains above elimination levels.

For covariate selection we used a simple, objective method. This is justified by our focus on spatial prediction rather than on the estimation of covariate effects per se, while also minimizing computational complexity. We employed correlation thresholds to exclude variables, which simplifies the selection process and is valid when assuming approximate linear relationships between covariates and the logit-transformed STH prevalence. Given the noisy nature of these empirical relationships, opting for a linear effect is a pragmatic choice, as the data do not support fitting more complex, non-linear models. Additionally, we relied on *p*-values to select statistically significant covariates at the 5% level. Although *p*-values are not a direct measure of a covariate's predictive strength, they offer a practical method for identifying variables that are most strongly associated with STH prevalence, thereby increasing the likelihood of improving model performance. Alternative metrics, such as the Akaike Information Criterion (AIC), Bayesian Information Criterion (BIC), or measures of predictive accuracy like cross-validated mean squared error (MSE), could be used for covariate selection. However, we do not anticipate that these alternative metrics would yield results remarkably different from those obtained using our current approach.

In the presence of residual spatial correlation, estimated covariate effects in observational studies of the kind considered here can be biased because of spatial confounding, which potentially occurs when a covariate d(x) is spatially correlated with the Gaussian process S(x). When comparing the estimates of the regression coefficients before after the inclusion of the Gaussian process S(x), we did find that this changed substantially, thus suggesting the presence of a non-negligible correlation between d(x) and S(x). This phenomenon, also known as spatial confounding, can lead to biased or inconsistent estimates of regression coefficients. Spatial confounding is particularly problematic when the primary objective is to draw causal or associative inferences on the regression coefficients. However, in our study, the focus is to identify useful spatial covariates that can improve spatial prediction of soil-transmitted helminth prevalence and classify areas into appropriate prevalence classes. Hence, the potential bias in the regression coefficients due to spatial confounding is less concerning, as long as the covariates contribute to better spatial prediction. In other words, we have not pursued unbiased estimation of individual covariate effects.

To measure socio-economic status (SES), studies have used variables such as land and farm animal ownership, the type of housing (rented or owner-occupied), level of education of the household head, demographic characteristics, and other proxy indicators for economy such as household head’s occupation [31]. In household and individual level surveys, such as the Demographic Health Survey, data on long-term asset ownership, access to utilities and infrastructure, as well as characteristics of housing are collected. One can use these factors to construct an SES index with principal components analysis [31]. However, the data on SES is only available at the sampled locations, and thus the use of an SES index as a predictor for STH prevalence would require the development of a bivariate geostatistical model. This could be used to infer the SES at unsampled locations whilst allowing for the propagation of the uncertainty into predictive inferences for prevalence. However, one of the issues of this approach is that the variation in SES can occur at fine spatial scale, making its use as a predictor for prevalence more difficult, especially when data are sparsely sampled over space. In this paper, we have considered proxies for SES that are available as raster files, such as poverty and WASH information. However, these proxies are modelled indicators and, as such, may not adequately capture the spatial heterogeneity inherent to SES.

This study highlights the significant impact of sparsely sampled data on uncertainty levels in classifying areas by endemicity. While covariates can help reduce this uncertainty, they may not suffice when very low uncertainty is required to inform critical decisions, such as continuing or stopping mass drug administration (MDA). Our analysis, based on site-level data from 23 counties in Kenya, lacks representation from certain regions, particularly in the north. Consequently, our results indicate that a substantial number of sub-counties cannot be classified if a certainty threshold below 80% is required.

To address this limitation, two strategies can be pursued: if resources allow, further data collection in unclassified regions could enhance classification accuracy by generating more precise probabilities. Alternatively, the probability threshold for classification could be lowered, though this would increase uncertainty. Using predictive probabilities to classify evaluation units provides a powerful framework for guiding targeted data collection, particularly in high-uncertainty scenarios where spatial units show similar probabilities across multiple prevalence classes, making reliable classification challenging. In these instances, the model identifies a lack of sufficient information to assign units confidently, enabling strategic allocation of resources to gather additional data where it is most needed. This focused approach optimizes sampling, thereby refining model predictions and enhancing the accuracy of prevalence maps, which are crucial for effective disease surveillance and control.

## Conclusions

This study demonstrates the substantial benefits of integrating spatially referenced covariates into geostatistical models for predicting the geographical distribution of STH. It emphasizes that these inclusions not only significantly enhance the model's performance but also improve the classification of regions into distinct prevalence classes, thereby offering a pathway to more targeted and effective MDA strategies. The robust performance of MBG models, as evidenced by simulation analyses, suggests that this approach remains effective even with smaller sample sizes and lower STH prevalence. This is especially important considering that as prevalence falls to very low levels following multiple rounds of MDA in many deworming programs, focal MDA campaigns, along with more innovative targeted approaches such as test and treat will be needed to achieve interruption of transmission.

## Supplementary Information

Additional file 1: Figure S1. Map of empirical logit - STH in Kenya. Figure S1a. Map of empirical logit - STH in Kenya by region. Table S1. Variables and the source. Table S1a. Covariates considered in different stages of selection for the final model. Table S2. Estimates and corresponding standard errors of the Binomial mixed model with unstructured random effects fitted with covariates to the STH prevalence data. Figure S2. Empirical variogram of the empirical logit of infection with any STH and theoretical variogram. Figure S3a. Scatter plots for empirical logit transformation against each of the selected covariates – Western region of Kenya. Figure S3b. Scatter plots for empirical logit transformation against each of the selected covariates – Eastern region of Kenya. Figure S3c. Map of covariates included in the geostatistical model and map of population density; Western region (top) and Eastern region (bottom). Figure S4. Standard error of predicted prevalence obtained from the geostatistical models without and with covariates in Western region of Kenya. Figure S5. Standard error of predicted prevalence obtained from the geostatistical models without and with covariates in Eastern region of Kenya. Table S3. Mean of log of standard errors of predicted prevalence of STH. Figure S6. Predictive probability maps for endemicity level between 10% and 20% for STH obtained from geostatistical models without and with covariates in Western region of Kenya. Figure S7. Predictive probability maps for endemicity level between 20% and 50% for STH obtained from geostatistical models without and with covariates in Western region of Kenya. Figure S8. Predictive probability maps for endemicity level greater than 50% for STH obtained from geostatistical models without and with covariates in Western region of Kenya. Figure S9. Predictive probability maps for endemicity level less than 2% for STH obtained from geostatistical models without and with covariates in Eastern region of Kenya. Figure S10. Predictive probability maps for endemicity level between 2% and 10% for STH obtained from geostatistical models without and with covariates in Eastern region of Kenya. Figure S11. Predictive probability maps for endemicity level between 10% and 20% for STH obtained from geostatistical models without and with covariates in Eastern region of Kenya. Figure S12. Predictive probability maps for endemicity level between 20% and 50% for STH obtained from geostatistical models without and with covariates in Eastern region of Kenya. Figure S13. Predictive probability maps for endemicity level greater than 50% for STH obtained from geostatistical models without and with covariates in Eastern region of Kenya. Figure S14. Proportion of correct classification and their differences at each sub-county for simulated dataset - sample size: 875; average prevalence: 12%. Figure S15. Proportion of correct classification and their differences at each sub-county for simulated dataset - sample size: 438; average prevalence: 6%. Figure S16. Proportion of correct classification and their differences at each sub-county for simulated dataset - sample size: 438; average prevalence: 12%. Figure S17. Proportion of correct classification and their differences at each sub-county for simulated dataset - sample size: 618; average prevalence: 10%. Figure S18. Proportion of correct classification and their differences at each sub-county for simulated dataset - sample size: 309; average prevalence: 1%. Figure S19. Proportion of correct classification and their differences at each sub-county for simulated dataset - sample size: 309; average prevalence: 10%.

## Data Availability

The datasets used and/or analysed during the current study are available from the corresponding author on reasonable request.

## Abbreviations

STH: Soil-transmitted helminthiasis
NSBDP: National school-based deworming programme
MDA: Mass drug administration
MBG: Model-based geostatistical
WHO: World Health Organization
MCML: Monte Carlo maximum likelihood
WASH: Water, Sanitation and Hygiene
SES: Socio-economic status
