# Supplementary material for: Understanding the impact of covariates on the classification of implementation units for soil-transmitted helminths control: a case study from Kenya
Source: BMC Med Res Methodol. 2024 Nov 29;24:294. doi: 10.1186/s12874-024-02420-1 (PMC11606136; doi:10.1186/s12874-024-02420-1)
Supplement: Supplementary file 1 — Additional file 1: Figure S1. Map of empirical logit - STH in Kenya. Figure S1a. Map of empirical logit - STH in Kenya by region. Table S1. Variables and the source. Table S1a. Covariates considered in different stages of selection for the final model. Table S2. Estimates and corresponding standard errors of the Binomial mixed model with unstructured random effects fitted with covariates to the STH prevalence data. Figure S2. Empirical variogram of the empirical logit of infection with any STH and theoretical variogram. Figure S3a. Scatter plots for empirical logit transformation against each of the selected covariates – Western region of Kenya. Figure S3b. Scatter plots for empirical logit transformation against each of the selected covariates – Eastern region of Kenya. Figure S3c. Map of covariates included in the geostatistical model and map of population density; Western region (top) and Eastern region (bottom). Figure S4. Standard error of predicted prevalence obtained from the geostatistical models without and with covariates in Western region of Kenya. Figure S5. Standard error of predicted prevalence obtained from the geostatistical models without and with covariates in Eastern region of Kenya. Table S3. Mean of log of standard errors of predicted prevalence of STH. Figure S6. Predictive probability maps for endemicity level between 10% and 20% for STH obtained from geostatistical models without and with covariates in Western region of Kenya. Figure S7. Predictive probability maps for endemicity level between 20% and 50% for STH obtained from geostatistical models without and with covariates in Western region of Kenya. Figure S8. Predictive probability maps for endemicity level greater than 50% for STH obtained from geostatistical models without and with covariates in Western region of Kenya. Figure S9. Predictive probability maps for endemicity level less than 2% for STH obtained from geostatistical models without and with covariates in Eastern region of Ke [file 12874_2024_2420_MOESM1_ESM.docx]

**Understanding the impact of covariates on the classification of implementation units for soil-transmitted helminths control: A case study from Kenya**

## Study site


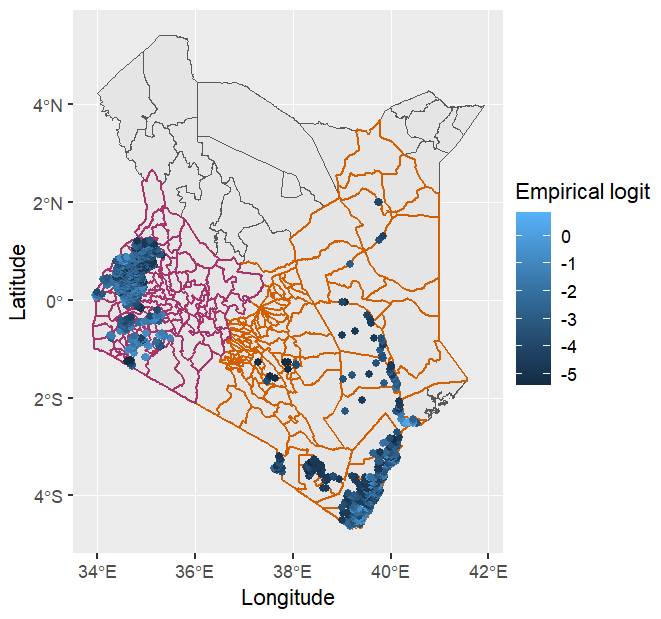


Figure S1: Map of empirical logit - STH in Kenya

To visualize the spatial coverage of the study region and to get an initial insight into the spatial pattern of prevalence, the empirical logit is used which is computed as

$$Empirical logit=log\left( \frac{num+0.5}{den-num+0.5} \right)$$

where, num=data on number of children tested and den= number positive of any STH.


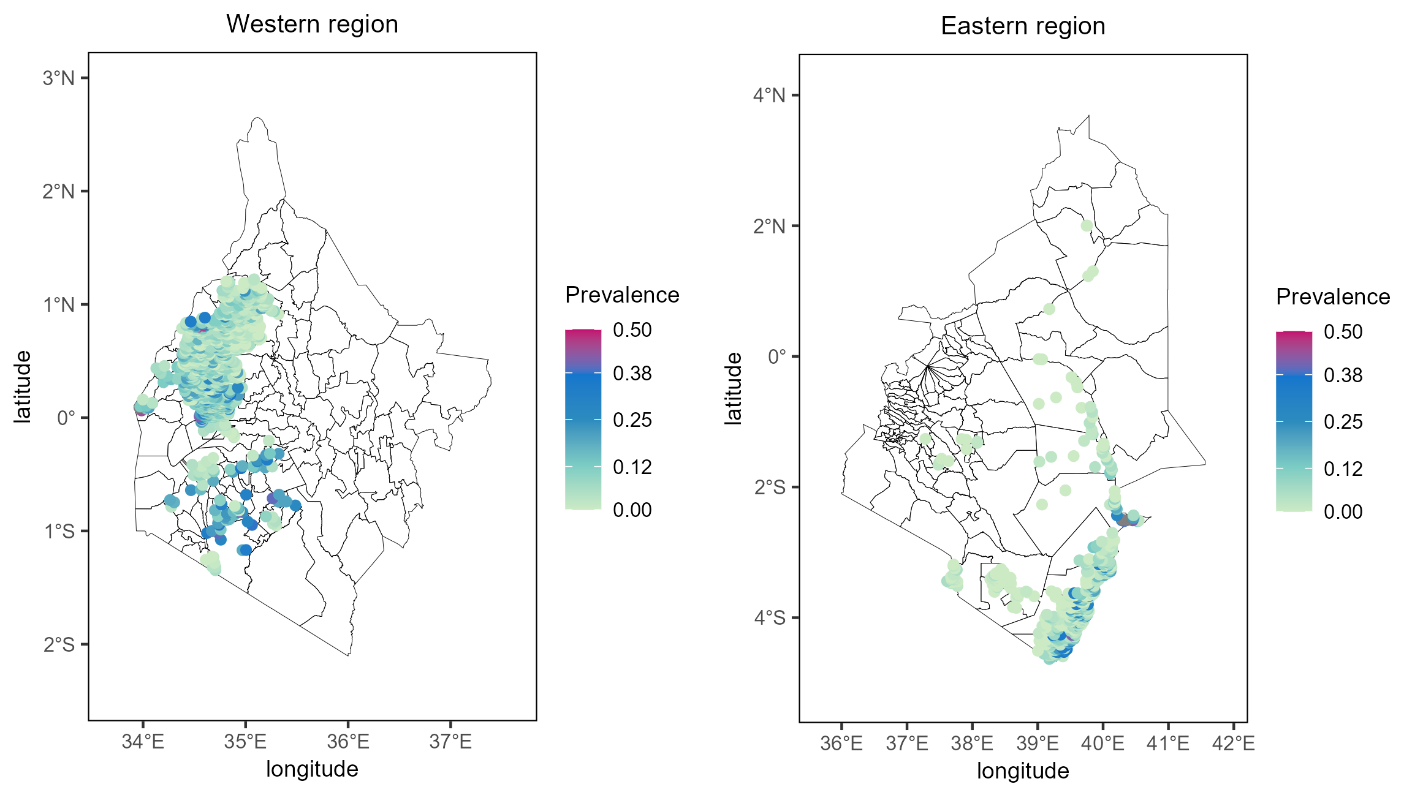


Figure S1a: Map of prevalence of STH in Kenya by region

## Variables and resources

Table S1: Variables and the source

| Variable | Source | Year | Approximate resolution |
| --- | --- | --- | --- |
| Aridity index | <http://www.cgiar-csi.org/wp-content/uploads/2012/11/Global-Aridity-and-Global-PET-Methodology.pdf> | annual average over the years 1970–2000 | 1km |
| Irrigated areas | <https://www.fao.org/aquastat/en/geospatial-information/global-maps-irrigated-areas> | 2005 | 10km |
| Potential evapotranspiration | <http://worldclim.org/> | annual average over the years 1970–2000 | 1km |
| Slope | <http://srtm.csi.cgiar.org/> | 2000 | 90m |
| WASH-availability of improved drinking water source | <https://www.thiswormyworld.org/maps/water-supply-and-sanitation-wss-coverage> | 2022 | 5km |
| WASH- availability of improved sanitation facility | <https://www.thiswormyworld.org/maps/water-supply-and-sanitation-wss-coverage> | 2022 | 5km |
| Wetlands | <http://due.esrin.esa.int/page_globcover.php#:~:text=GlobCover%20is%20an%20ESA%20initiative,board%20the%20ENVISAT%20satellite%20mission> | 2015 | 5km |
| Urban areas-Grump | <https://sedac.ciesin.columbia.edu/data/set/grump-v1-urban-extents> | 2015 | 5km |
| Elevation | <http://viewfinderpanoramas.org/dem3.html> | 2021 | 90m |
| Improved housing prevalence | <https://www.nature.com/articles/s41586-019-1050-5> | 2015 | 5km |
| Soil moisture | <https://cds.climate.copernicus.eu/cdsapp#!/dataset/satellite-soil-moisture?tab=overview> | 2022 | 1km |
| Poverty | <https://www.worldpop.org/geodata/summary?id=1262> | 2020 | 1km |
| Night lights | <https://eogdata.mines.edu/download_dnb_composites.html> | 2020 | 1km |
| Rainfall - monthly average | <https://www.chc.ucsb.edu/data/chirps> | 2020-2021 |  |
| Enhanced vegetation index - monthly average | <http://modis.gsfc.nasa.gov/data/dataprod/dataproducts.php?MOD_NUMBER=13> | 2020 | 1km |
| Land surface temperature - Day - monthly average | <https://lpdaac.usgs.gov/products/mod11a1v006/> | 2020 | 1km |
| Land surface temperature - Night - monthly average | <https://lpdaac.usgs.gov/products/mod11a1v006/> | 2020 | 1km |
| Land surface temperature - Difference - monthly average | <https://lpdaac.usgs.gov/products/mod11a1v006/> | 2020 | 1km |
| Tasseled cap brightness - monthly average | <https://lpdaac.usgs.gov/dataset_discovery/modis/modis_products_table/mcd43b4> | 2020 | 1km |
| Tasseled cap wetness - monthly average | <https://lpdaac.usgs.gov/dataset_discovery/modis/modis_products_table/mcd43b4> | 2020 | 1km |
| Population - interpolated | <https://www.worldpop.org/geodata/listing?id=64,https://sedac.ciesin.columbia.edu/data/collection/gpw-v4> | 2020 | 1km |
| Urban area-Building Density | <https://sites.research.google/open-buildings> | 2021 | 5km |
| Grasslands | <https://lpdaac.usgs.gov/products/mcd12q1v006/> | 2020 | 1km |
| Croplands | <https://lpdaac.usgs.gov/products/mcd12q1v006/> | 2020 | 1km |
| Croplands / Natural vegetation | <https://lpdaac.usgs.gov/products/mcd12q1v006/> | 2020 | 1km |

Table S1a: Covariates considered in different stages of selection for the final model

| Covariates considered | Covariates considered after removing highly correlated ones | Covariates considered after removing those poorly correlated with empirical logit of STH prevalence | Covariates considered in logistic regression model | Covariates considered in binomial geostatistical model |
| --- | --- | --- | --- | --- |
| Aridity index | Aridity index | Aridity index | Aridity index | Aridity index |
| Irrigated areas | Irrigated areas | Water | Water | Water |
| Potential evapotranspiration | Potential evapotranspiration | Elevation | Elevation | Elevation |
| Slope | Water | Rainfall | Rainfall | Rainfall |
| WASH-availability of improved drinking water source (Water) | Sanitation | EVI | EVI | EVI |
| WASH- availability of improved sanitation facility  (Sanitation) | Elevation | TCW | TCW | TCW |
| Wetlands | Soil moisture |  |  |  |
| Elevation | Poverty |  |  |  |
| Improved housing prevalence | Rainfall - monthly average |  |  |  |
| Soil moisture | EVI |  |  |  |
| Poverty | LST-Day |  |  |  |
| Night lights | LST-Difference |  |  |  |
| Rainfall - monthly average | TCB |  |  |  |
| Enhanced vegetation index - monthly average (EVI) | TCW |  |  |  |
| Land surface temperature (LST) - Day - monthly average | Urban area-Building Density |  |  |  |
| Land surface temperature (LST) - Night - monthly average | Grasslands |  |  |  |
| Land surface temperature (LST) - Difference - monthly average | Croplands |  |  |  |
| Tasseled cap brightness - monthly average (TCB) |  |  |  |  |
| Tasseled cap wetness - monthly average (TCW) |  |  |  |  |
| Urban area-Building Density |  |  |  |  |
| Grasslands |  |  |  |  |
| Croplands |  |  |  |  |
| Croplands / Natural vegetation |  |  |  |  |

## Logistic regression results

Table S2: Estimates and corresponding standard errors of the binomial logistic regression model fitted with covariates to the STH prevalence data

| Region |  | Estimate | Standard error | p value |
| --- | --- | --- | --- | --- |
| Kenya-West | Intercept | -1.223 | 0.174 | 2.06e-12 |
|  | Aridity | 0.985 | 0.127 | 9.10e-15 |
|  | Enhanced vegetation index | 4.381 | 0.383 | <2e-16 |
|  | Tassled cap wetness | 12.734 | 0.665 | <2e-16 |
|  | Availability of drinking water | -1.883 | 0.096 | <2e-16 |
| Kenya-East | Intercept | -5.997 | 0.185 | <2e-16 |
|  | Aridity | 3.282 | 0.304 | <2e-16 |
|  | Enhanced vegetation index | 6.682 | 0.365 | <2e-16 |
|  | Rainfall | -0.014 | 0.002 | <2e-16 |
|  | Elevation | -0.002 | 0.00014 | <2e-16 |

## Testing for residual spatial variation

To establish whether prediction of prevalence throughout the study-region would benefit from geostatistical methods, empirical variogram is calculated. The empirical variogram is useful for establishing that the data show evidence of spatial correlation and for suggesting a suitable form of parametric model. The plot (Figure 2) suggested that the empirical variogram levels out, indicating that the spatial correlation becomes negligible, at a distance somewhere in the range 100 to 120 kms and at a distance of 200 to 400 kms in the western region and eastern region, respectively.

**
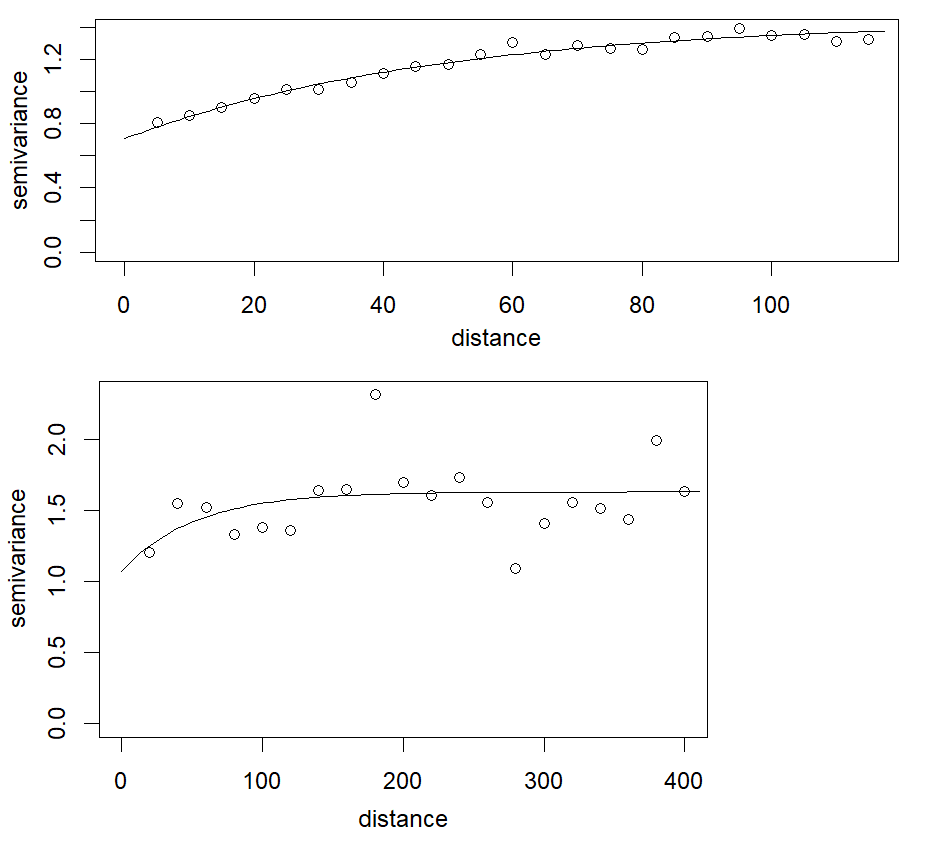
**

Figure S2: Empirical variogram of the empirical logit of infection with any STH (open circles) and a theoretical variogram of the form $V(u) = \tau^{2} +\sigma^{2}(1-exp(-u/\varphi)$*,* where u is distance (km) – Western region of Kenya (top) and Eastern region of Kenya (bottom)

## Scatter plots for exploratory analysis and map of covariates

The simple linear fit is depicted by the dashed green line and the solid blue line represents the natural spline.


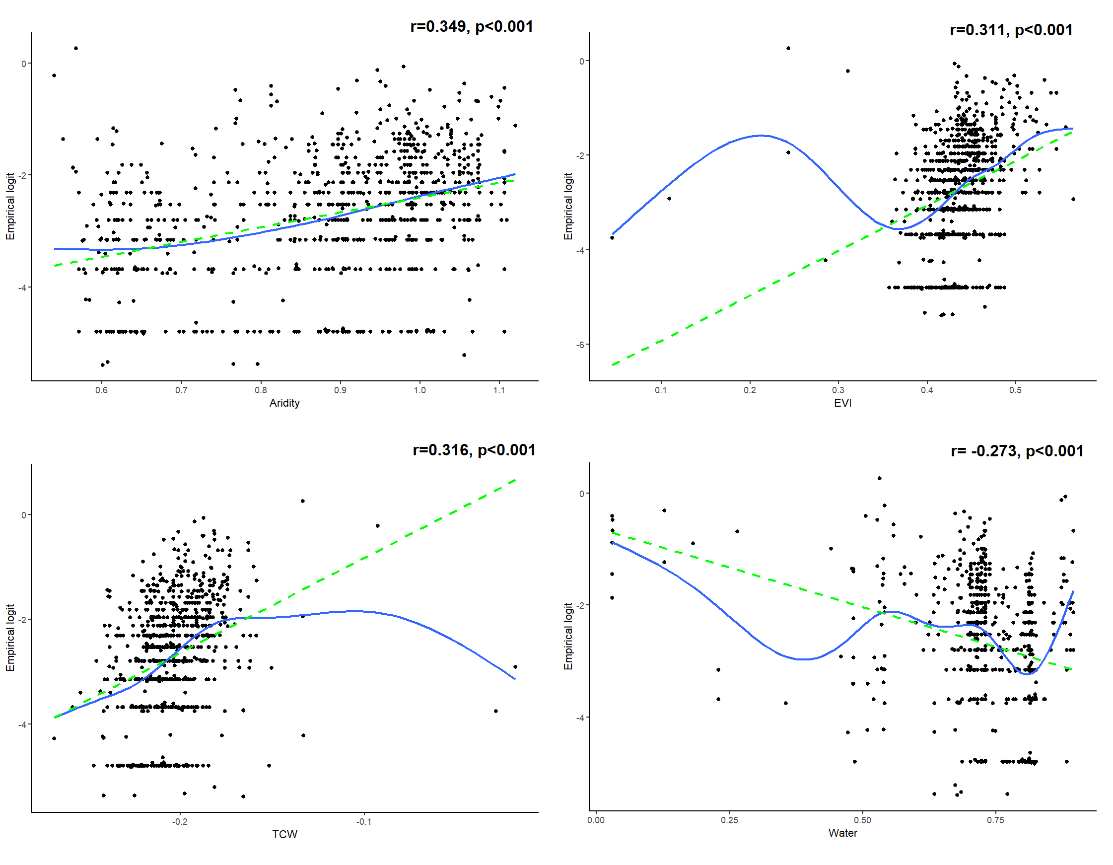


Figure S3a: Scatter plots for empirical logit transformation against each of the selected covariates – Western region of Kenya


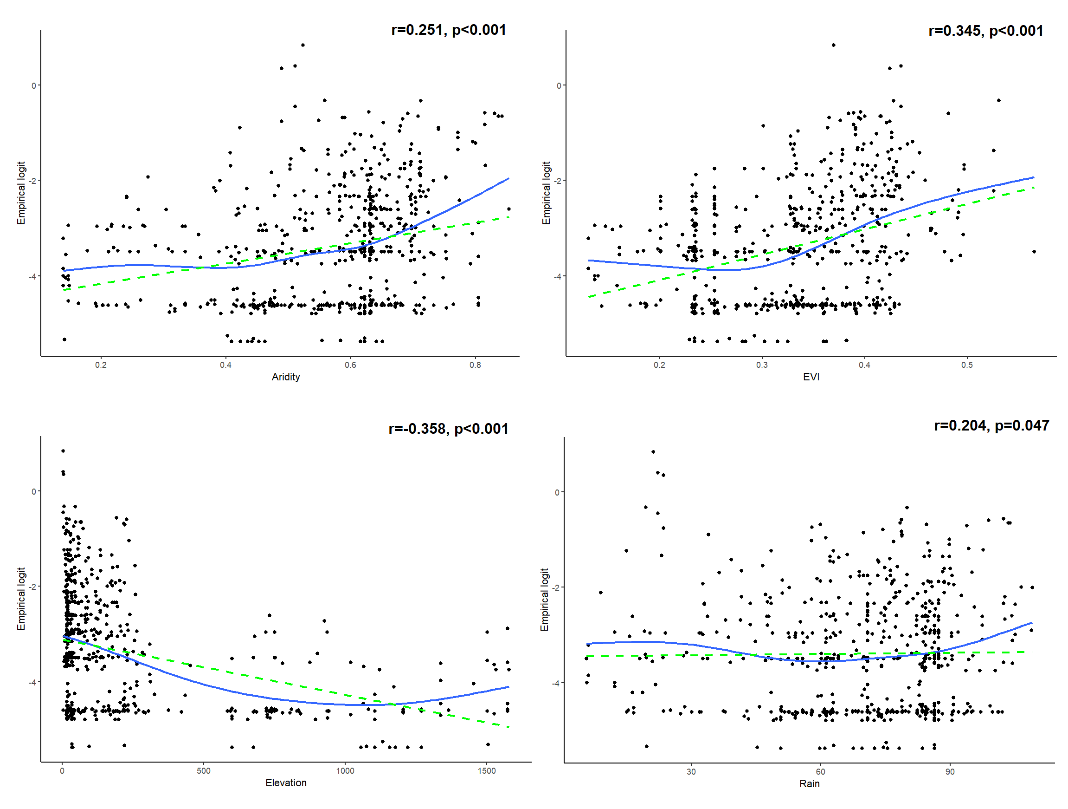


Figure S3b: Scatter plots for empirical logit transformation against each of the selected covariates – Eastern region of Kenya


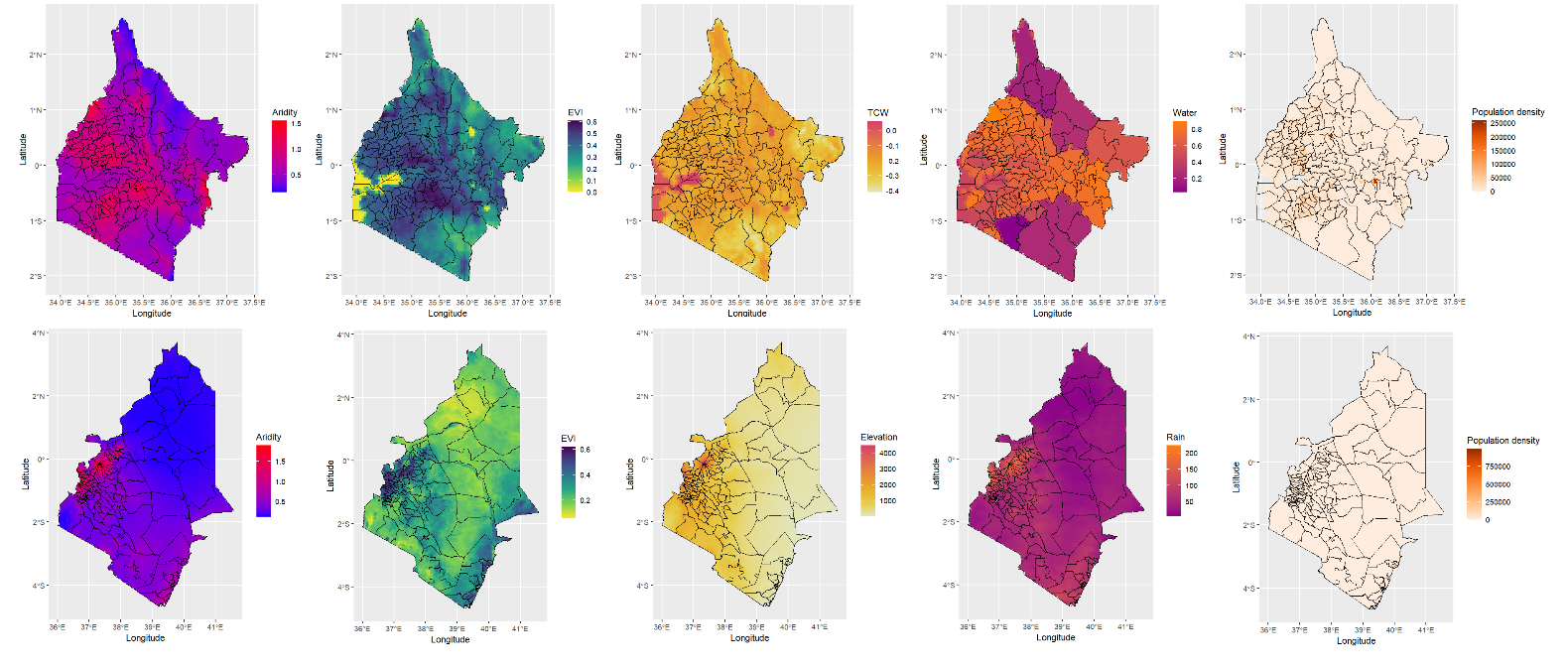


Figure S3c: Map of covariates included in the geostatistical model and map of population density; Western region (top) and Eastern region (bottom)

## Mapping of standard error of predicted prevalence


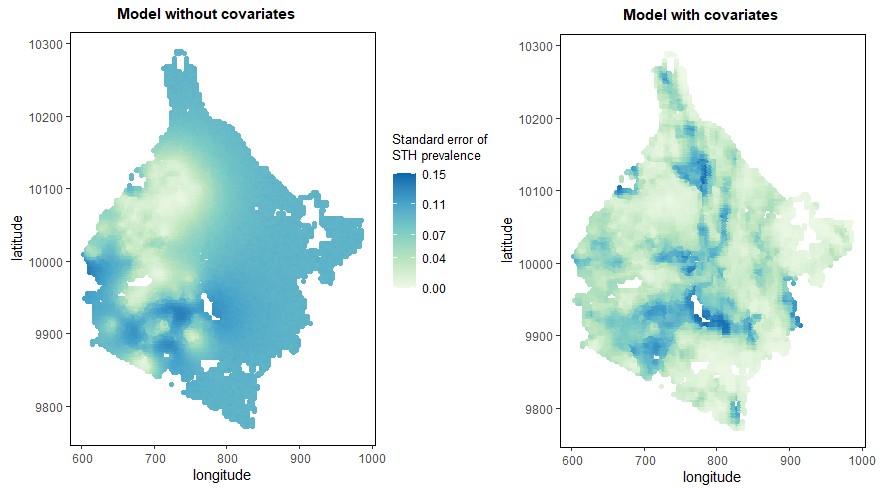


Figure S4: Standard error of predicted prevalence obtained from the geostatistical intercept only model and model with covariates in Western region of Kenya


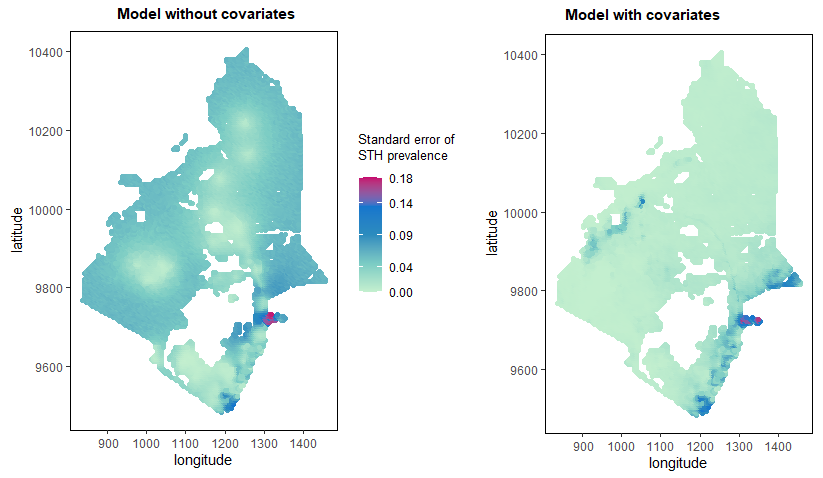


Figure S5: Standard error of predicted prevalence obtained from the geostatistical intercept only model and model with covariates in Eastern region of Kenya

The predictive precision from the two models were compared using the mean of log of standard errors of predicted prevalence given in Table S3. It was observed that the model with covariates in both regions provides increased precision.

Table S3: Mean of log of standard errors of predicted prevalence of STH

| Model | Mean log of standard errors | |
| --- | --- | --- |
|  | Western region | Eastern region |
| Intercept only | -2.655 | -3.253 |
| With covariates | -3.450 | -4.876 |

## Predictive probability maps


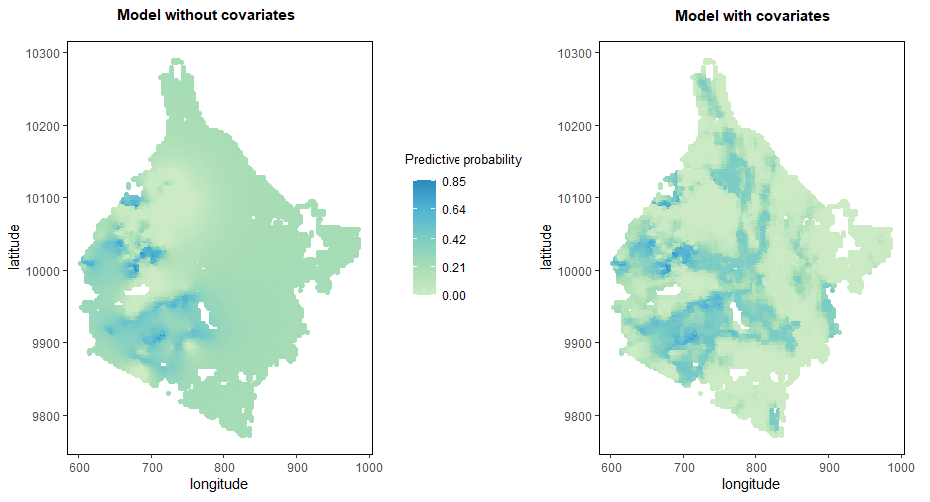


Figure S6: Predictive probability maps for endemicity level between 10% and 20% for STH obtained from geostatistical intercept only model in the Western region of Kenya


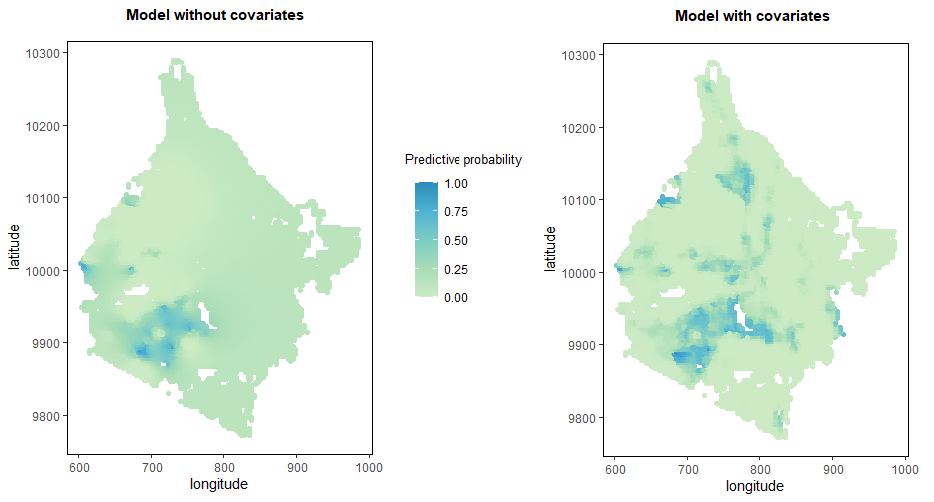


Figure S7: Predictive probability maps for endemicity level between 20% and 50% for STH obtained from geostatistical intercept only model in the Western region of Kenya


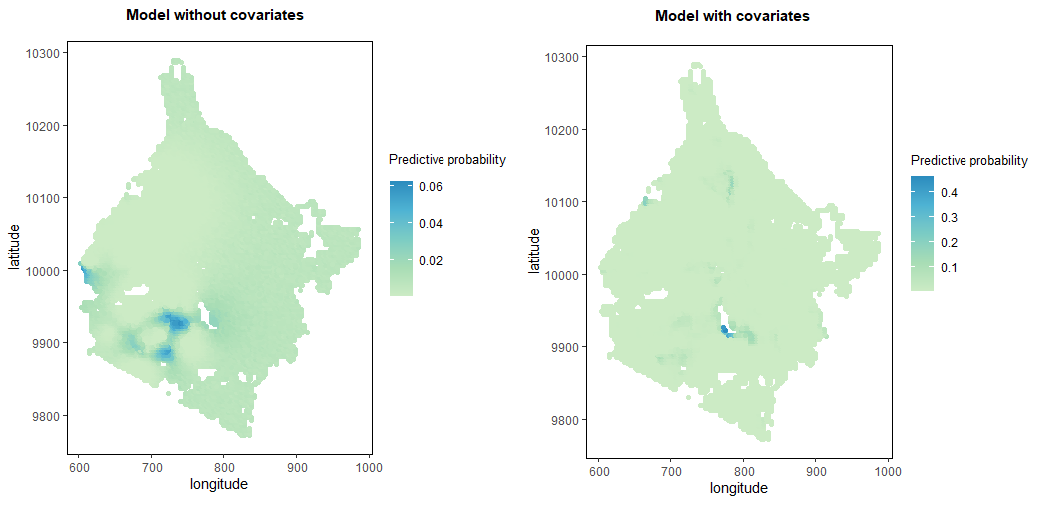


Figure S8: Predictive probability maps for endemicity level greater than 50% for STH obtained from geostatistical intercept only model in the Western region of Kenya


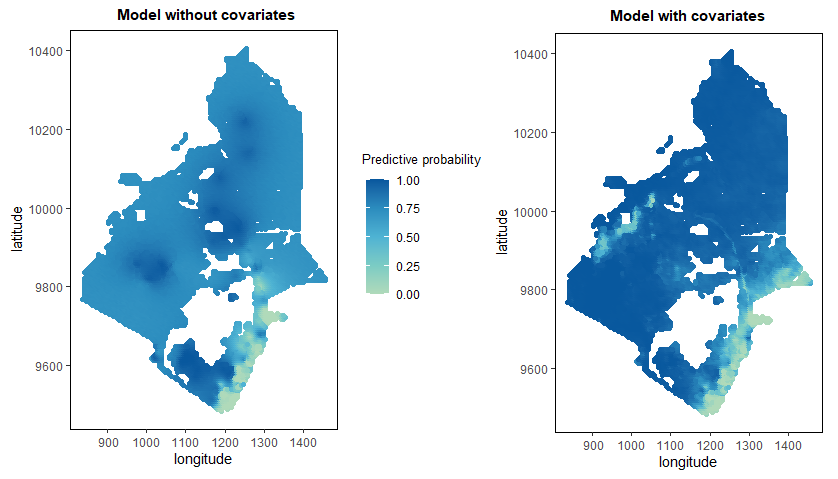


Figure S9: Predictive probability maps for endemicity level less than 2% for STH obtained from geostatistical intercept only model in the Eastern region of Kenya


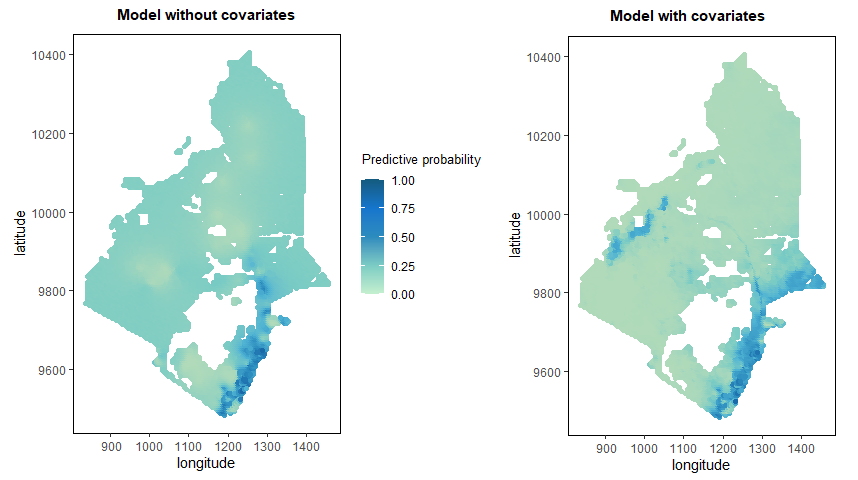


Figure S10: Predictive probability maps for endemicity level between 2% and 10% for STH obtained from geostatistical intercept only model in the Eastern region of Kenya


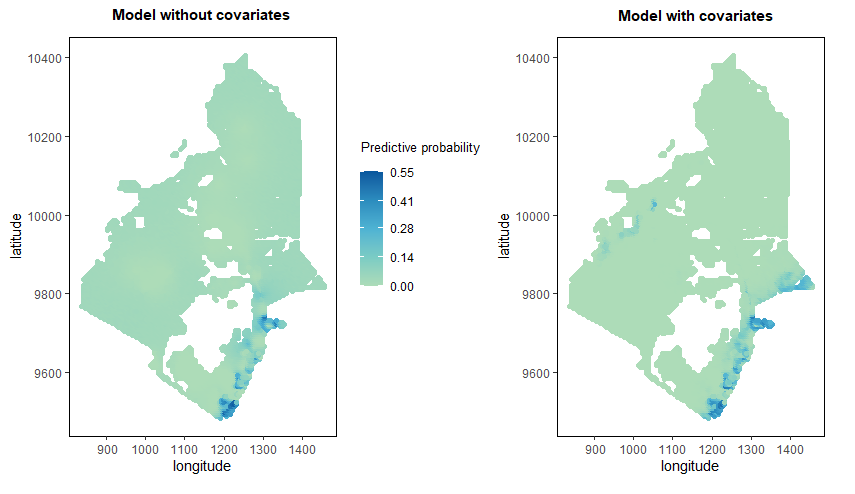


Figure S11: Predictive probability maps for endemicity level between 10% and 20% for STH obtained from geostatistical intercept only model in the Eastern region of Kenya


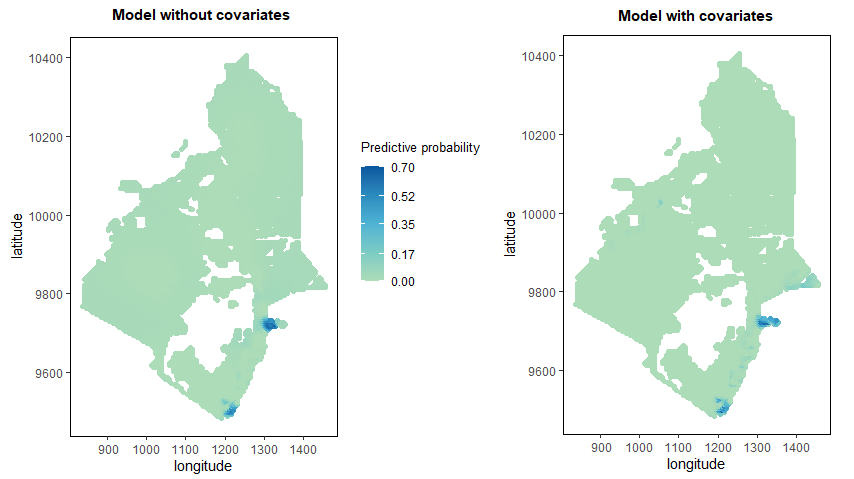


Figure S12: Predictive probability maps for endemicity level between 20% and 50% for STH obtained from geostatistical intercept only model in the Eastern region of Kenya


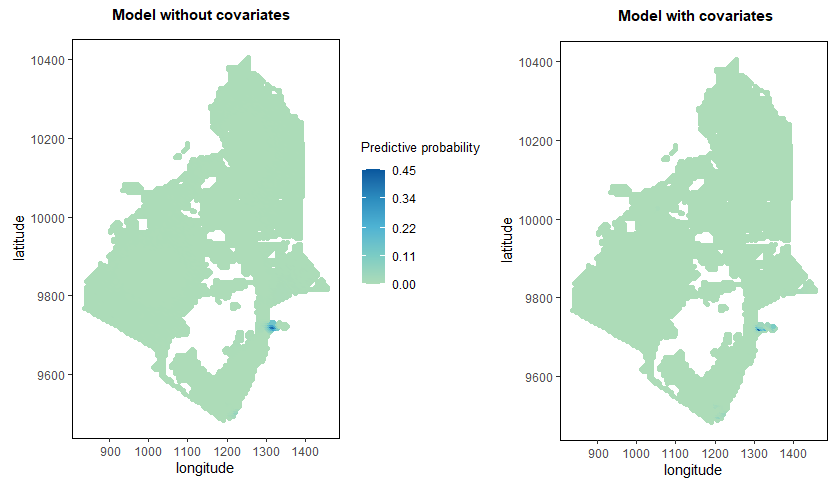


Figure S13: Predictive probability maps for endemicity level greater than 50% for STH obtained from geostatistical intercept only model in the Eastern region of Kenya

## Simulation study

1. Maps of simulation study results in the Western region


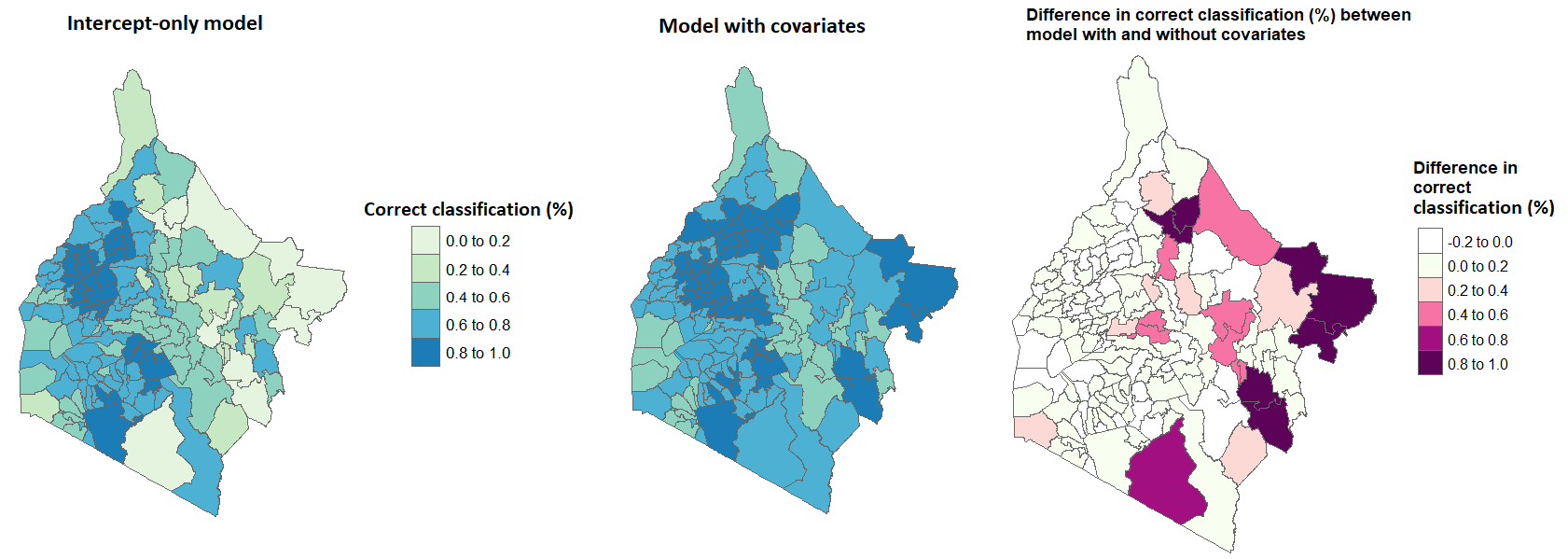


Figure S14: Proportion of correct classification and their differences at each sub-county for simulated dataset with sample size of 875 and average prevalence of 12%, using the sampling technique of observed data


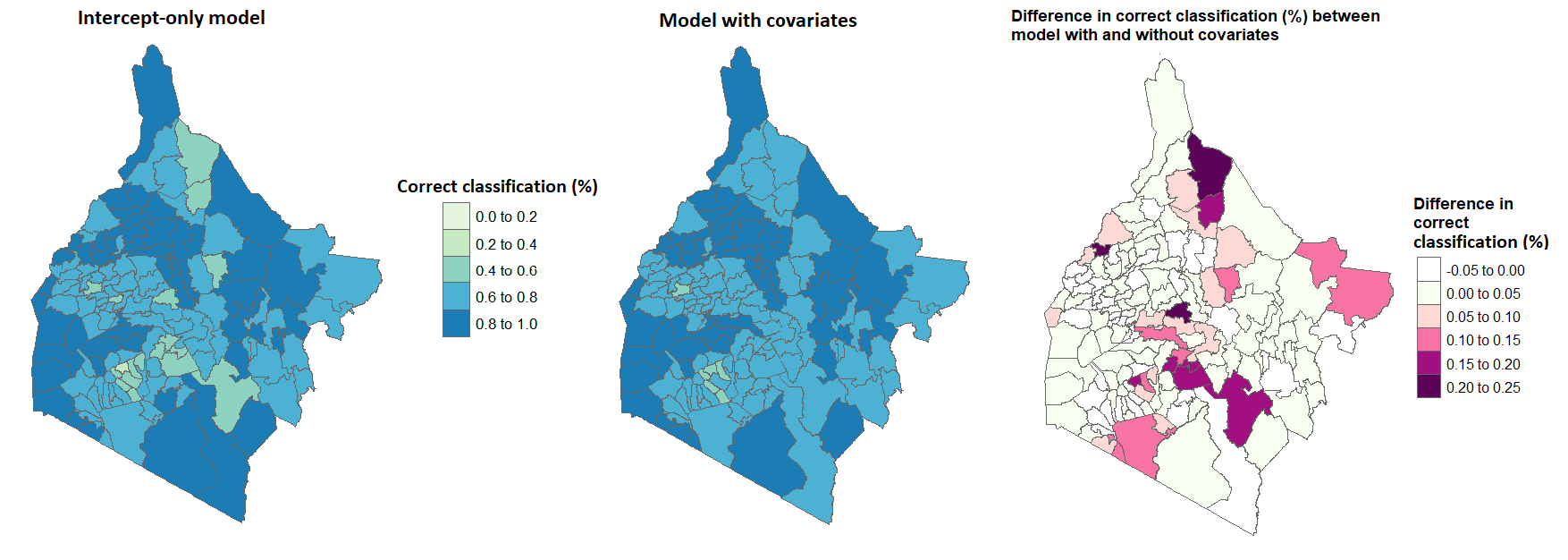


Figure S15: Proportion of correct classification and their differences at each sub-county for simulated dataset with sample size of 438 and average prevalence of 6%, using the sampling technique of observed data


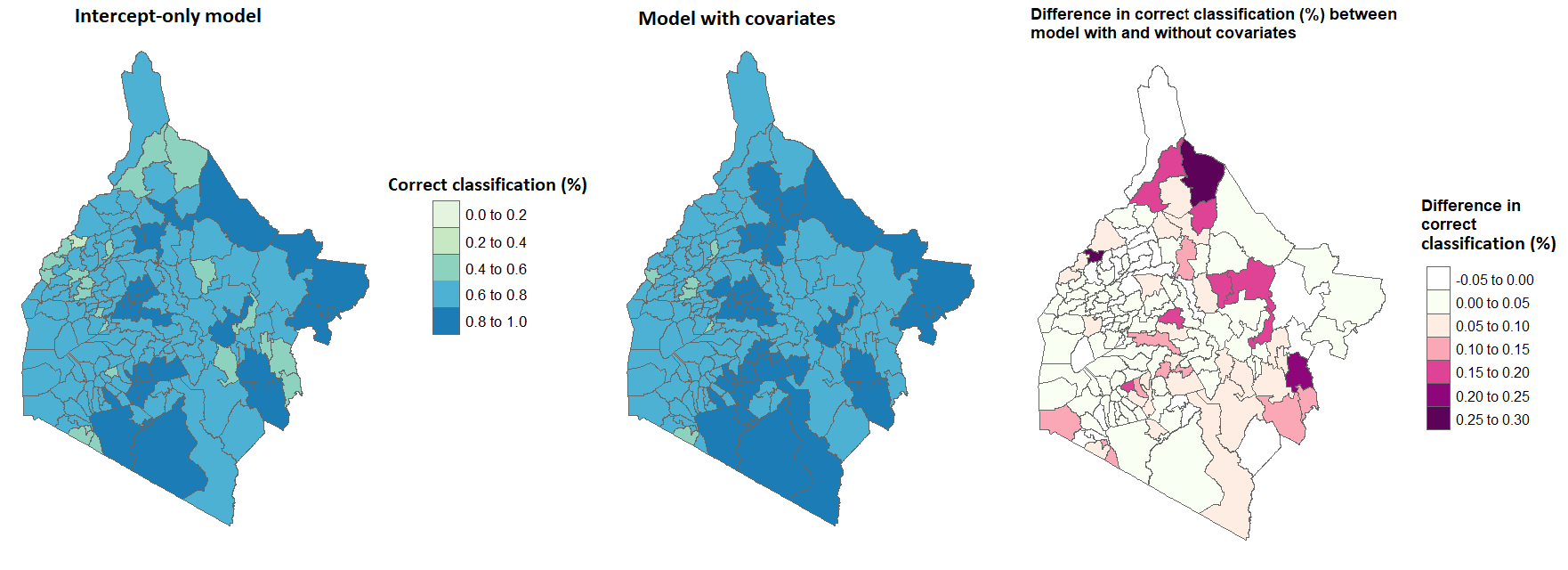


Figure S16: Proportion of correct classification and their differences at each sub-county for simulated dataset with sample size of 438 and average prevalence of 12%, using the sampling technique of observed data

1. Maps of simulation study results in the Eastern region


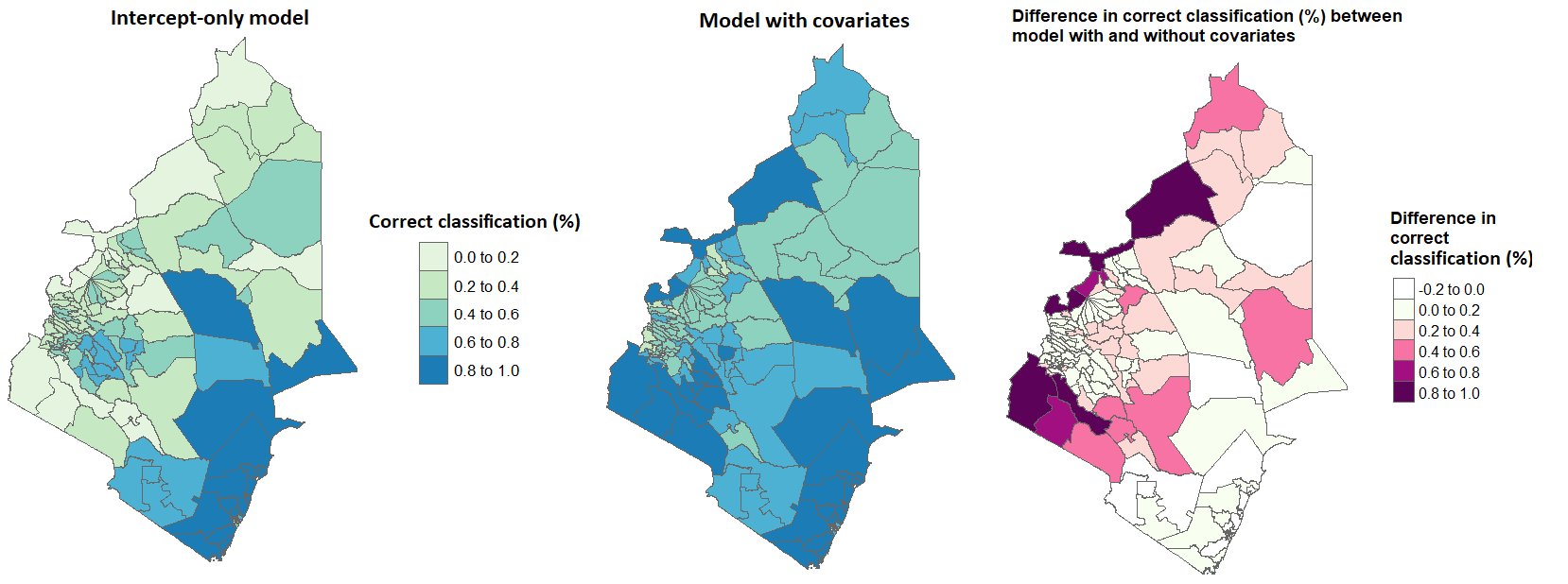


Figure S17: Proportion of correct classification and their differences at each sub-county for simulated dataset with sample size of 618 and average prevalence of 10%, using sampling technique of observed data


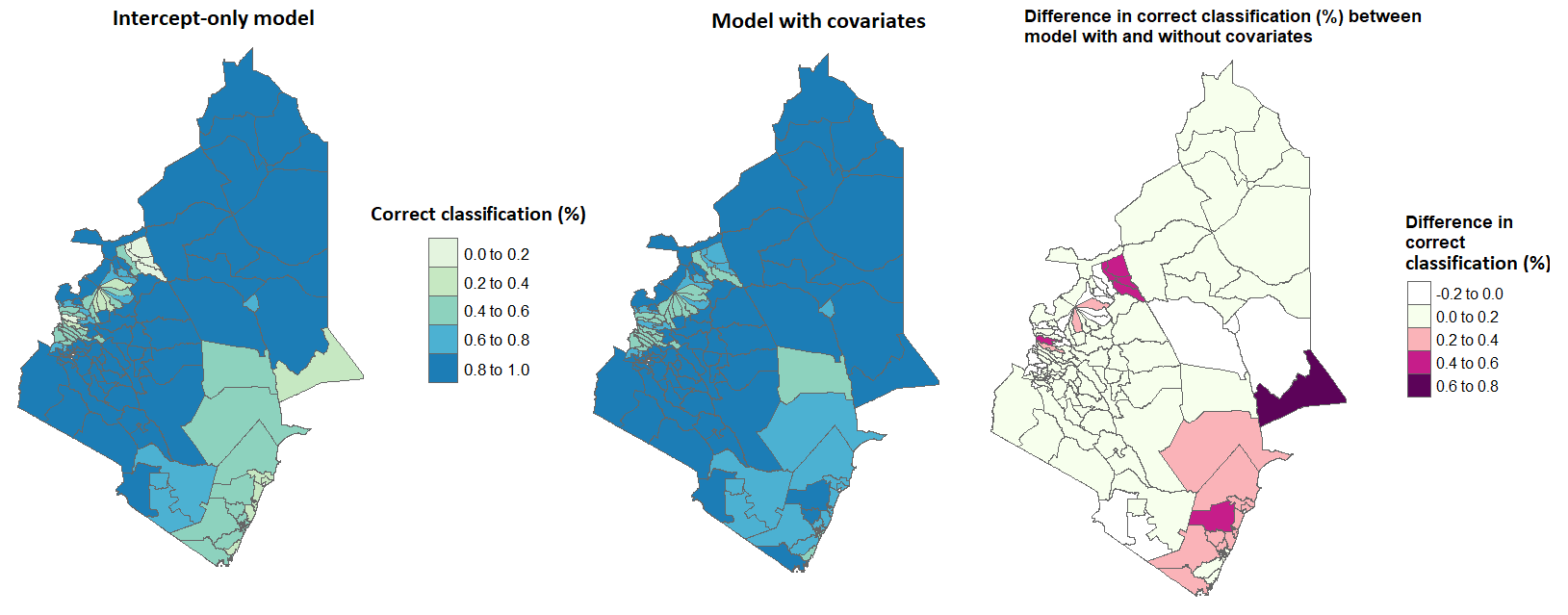


Figure S18: Proportion of correct classification and their differences at each sub-county for simulated dataset with sample size of 309 and average prevalence of 1%, using the sampling technique of observed data


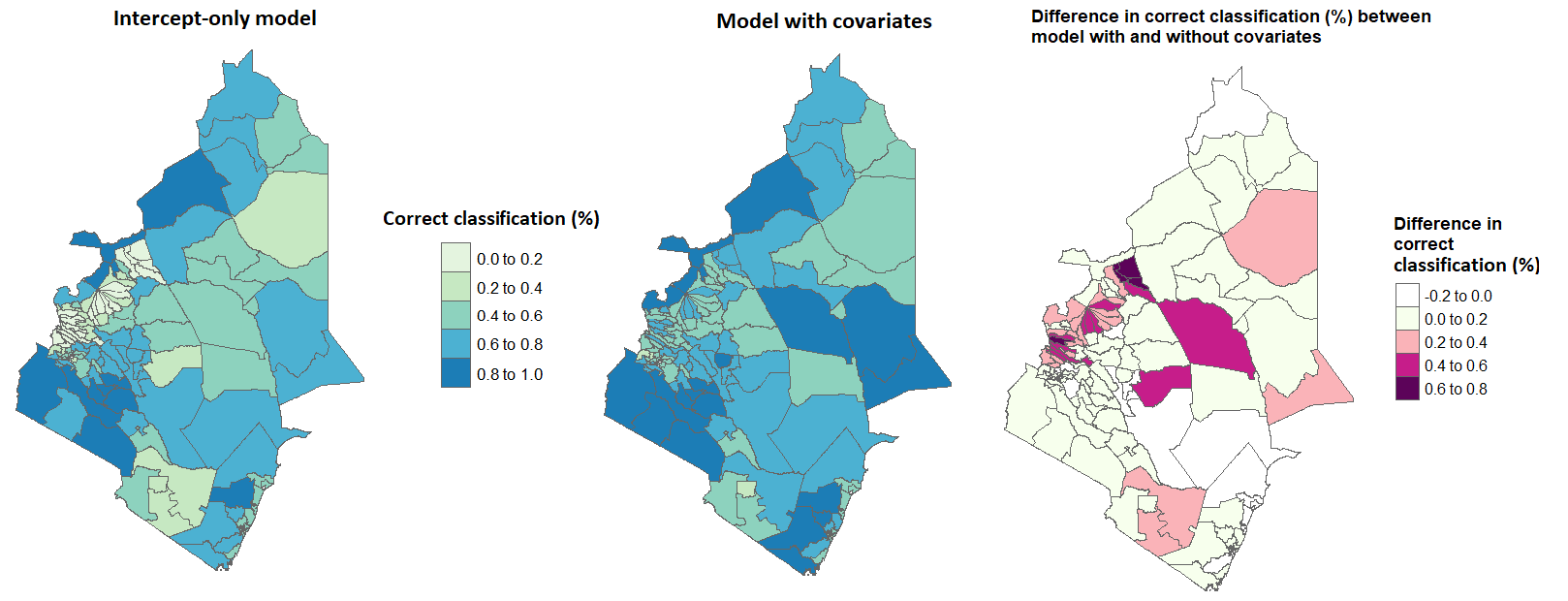


Figure S19: Proportion of correct classification and their differences at each sub-county for simulated dataset with sample size of 309 and average prevalence of 10%, using the sampling technique of observed data
